# Development of a brain-permeable peptide nanofiber that prevents aggregation of Alzheimer pathogenic proteins

**DOI:** 10.1371/journal.pone.0235979

**Published:** 2020-07-24

**Authors:** Naoki Tanaka, Michiaki Okuda, Tatsutoshi Nishigaki, Nobuhiko Tsuchiya, Yukako Kobayashi, Takuya Uemura, Sayaka Kumo, Hachiro Sugimoto, Seiji Miyata, Tomonori Waku

**Affiliations:** 1 Faculty of Molecular Chemistry and Engineering, Kyoto Institute of Technology, Kyoto, Japan; 2 Department of Pharmacology, Graduate School of Pharmaceutical Sciences, Kyoto University, Kyoto, Japan; 3 Faculty of Life and Medical Sciences, Doshisha University, Kyoto, Japan; 4 Faculty of Applied Biology, Kyoto Institute of Technology, Kyoto, Japan; National Center for Geriatrics and Gerontology, JAPAN

## Abstract

Alzheimer's disease (AD) is proposed to be induced by abnormal aggregation of amyloidβ in the brain. Here, we designed a brain-permeable peptide nanofiber drug from a fragment of heat shock protein to suppress aggregation of the pathogenic proteins. To facilitate delivery of the nanofiber into the brain, a protein transduction domain from *Drosophila* Antennapedia was incorporated into the peptide sequence. The resulting nanofiber efficiently suppressed the cytotoxicity of amyloid βby trapping amyloid β onto its hydrophobic nanofiber surface. Moreover, the intravenously or intranasally injected nanofiber was delivered into the mouse brain, and improved the cognitive function of an Alzheimer transgenic mouse model. These results demonstrate the potential therapeutic utility of nanofibers for the treatment of AD.

## Introduction

Alzheimer’s disease (AD) is a progressive neurodegenerative disorder and the most common form of dementia [1] characterized by senile plaque (SP), which is the extracellular deposit of amyloidβ(Aβ)aggregates [2],and neurofibrillary tangle, which is the intracellular accumulation of phosphorylated tau protein [3]. Although highly ordered linear Aβaggregates, known as amyloid fibrils [4, 5], are the major component of SP and one of the hallmark features of AD, soluble oligomers of aggregates are regarded as more cytotoxic [6]. The therapeutic use of peptide inhibitors of pathological aggregation has been proposed for the treatment of AD [7, 8]. Synthetic peptides from α-crystallin have been extensively examined for inhibiting pathological aggregation, as fragments of this protein are unlikely to induce an immune response [9–11]. α-Crystallin, a member of the small heat shock superfamily of proteins, can prevent protein aggregation [9, 12–16]. The α-crystallin protein consists of two closely related subunits, A and B (20 kDa each), and displays a β-sheet rich structure [17]. Synthetic peptides of α-crystallin corresponding to the substrate binding regions of the A and B subunits also inhibit aggregation of various proteins, including citrate synthase, alcohol dehydrogenase, and insulin [18–27]. In 2004, Santhosh kumar reported pioneering research indicating the possibility of a peptide derived from the substrate binding site of the A subunit of α-crystallin, DFVIFLDVKHFSPEDLTVK, as a drug against AD. The authors showed that the peptides inhibit fibril formation of Aβ peptides and suppress the toxicity of Aβin rat pheochromocytoma (PC12) [19]. Subsequently, the same research group reported that the cell-penetrating peptide-fused type peptides (DFVIFLDVKHFSPEDLTVK, CPP-fused αAC peptides) also prevented Aβ fibril formation and suppress Aβ toxicity [23].

We previously established that a peptide of the substrate binding site of the α-crystallin A subunit, comprising amino acid residues 71–88 (αAC(71–88), FVIFLDVKHFSPEDLTVK),forms a β-sheet rich nanofiber (αAC nanofiber) with the negatively charged surface [28, 29]. The activity of αAC(71–88) to suppress protein aggregation is enhanced by nanofiber formation, because a hydrophobic environment is generated on the nanofiber surface that traps the small protein aggregates. Thus, the αAC nanofibers could be a candidate substance for the treatment of AD. However, while the aggregation of Aβ (1–40) was suppressed by the αAC nanofiber, that of the fragment comprising the three-repeats of a microtubule-binding domain (3RMBD) of tau protein was promoted; this is remarkably unfavorable for developing the therapeutic peptide nanofibers for the treatment of AD because both proteins are AD-related [29]. The apparent surface charge of substrate proteins is the key factor determining whether protein aggregation is suppressed or facilitated by αAC nanofiber [29]; the negatively charged αAC nanofibers act as an inhibitor of the aggregation of anionic proteins, including Aβ (1–40) (pI = 5.5),whereas they facilitate the aggregation of cationic proteins, including 3BMBD of tau (pI = 9.6) [30, 31]. Thus, when targeting the aggregation of Aβ and tau, charged nanofibers are problematic as they can suppress the aggregation of one protein, while promoting the aggregation of the other. Therefore, we hypothesized that charge neutralization of the nanofiber surface could be the solution to this issue.

**Fig 1:**
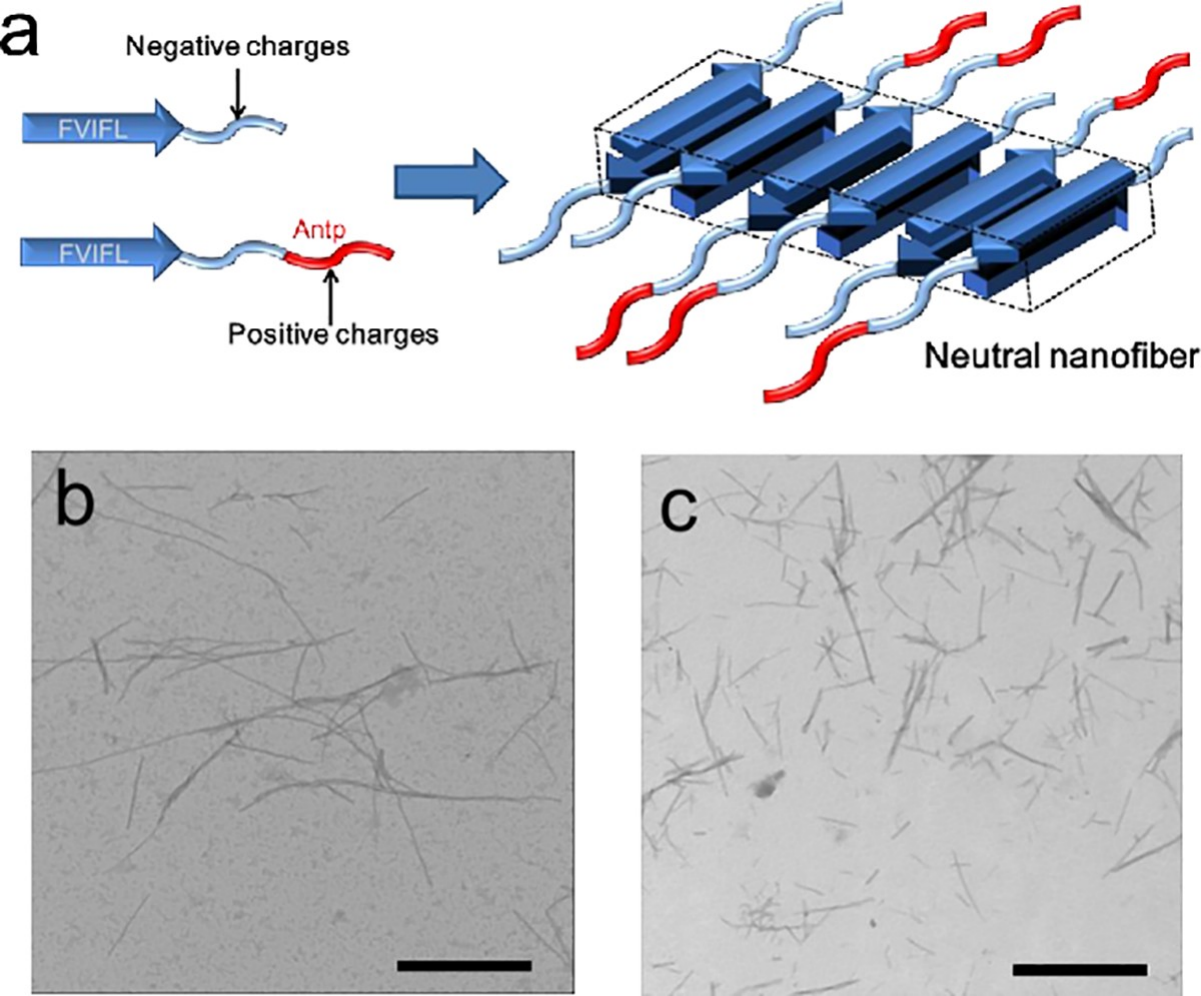
Structure of the αAC nanofiber. (a) Schematic illustration of the neutral AC nanofibers composed of αAC(71–88) peptide and αAC(71–88)Antp peptide. The hydrophilic segments would be outside of the nanofiber core. The proposal structures of the neutral αAC nanofibers are based on our previous work, which reported the structural analysis of the peptide nanofibers formed from amphiphilic peptides similar to the αAC(71–88)Antp peptides. (b) TEM image of the nanofibers prepared by incubation of αAC(71–88)Antp obtained by incubation of 1 mg/ml peptide in 5 mM phosphate pH 7.5, 100 mM NaCl with 10% HFIP at 60°Cfor 24 hr. (c) TEM image of the nanofibers prepared by incubation of a solution containing αAC(71–88) and αAC(71–88)Antp (molar ratio; 4:1). The scale bars represent 1 μm. The nanofiber solutions were centrifuged and the resulting pellet was resuspended in 5 mM phosphate buffer containing 100 mM NaCl (pH 7.5). Finally, the solution was dialyzed against the same buffer to completely remove HFIP.

In the present study, we developed a unique nanofiber type peptide drug from the fragments of α-crystallin for the treatment of AD. We designed an electrostatically neutralαAC nanofiber, composed of αAC(71–88) and its cationic variant that are fused with a protein transduction domain from *Drosophila* Antennapedia (Antp; RQIKIWFQNRRMKWKK) (Fig 1A). Antp is a peptide sequence that enhances cellular transduction, even of very large particles [32], and thereby facilitates the delivery of intravenously-injected therapeutic peptides into the brain across the blood-brain barrier (BBB) [33]. Moreover, recent research reported that the delivery efficiency of proteins into the brain were enhanced by intranasal coadministration of Antp peptides [34]. These findings suggest that the introduction of Antp sequences into nanofibers allows them to reach the brain via both administration routes.

## Results

### Properties of αAC(71–88)Antp nanofiber

At first,we examined nanofiber formation of a cationic variant of αAC(71–88) that was fused to a protein transduction domain from *Drosophila* Antennapedia i.e., αAC(71–88)Antp (FVIFLDVKHFSPEDLTVKRQIKIWFQNRRMKWKK). The nanofiber shown in Fig 1B was obtained by incubation of a 1 mg/ml solution of peptide in 5 mM phosphate (pH 7.5), 100 mM NaCl and 10% hexafluoro isopropanol(HFIP) at 60°C. Far-UV CD spectra indicated that the α-helix rich conformation of αAC(71–88)Antp changed to a β-sheet rich profile (S1 Fig in S1 File) upon nanofiber formation, indicating that its structure is similar to that of the amyloid fibril. The zeta potential value of the αAC(71–88)Antp nanofiber was ca. + 40 mV, suggesting the presence of a positively charged surface. The fluorescence emission of 1-anilino-8-naphthalene sulfonate (ANS) bound to the αAC(71–88)Antp nanofiber exhibited a concomitant blue shift of its maximum fluorescence emission with increased fluorescence intensity (S2 Fig in S1 File). Therefore, the surface of the αAC(71–88)Antp nanofiber comprises a highly hydrophobic environment, which is suitable for substrate binding.

**Fig 2:**
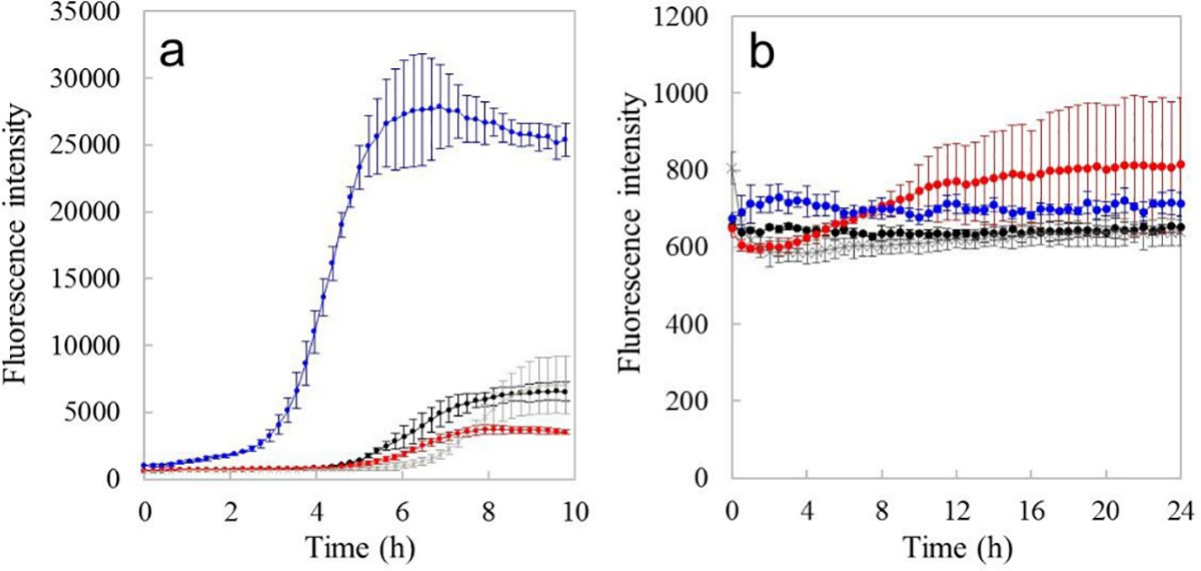
The effect of the nanofibers made up of derivatives of αAC(71–88) on the aggregation of the pathological proteins of Alzheimer’s disease was monitored by analyzing ThT fluorescence. (a)Time course of ThT fluorescence intensity of 10μg/ml Aβ (1–42) solutions incubated at 37°C. Black plot, Aβ (1–42) alone; blue plot, + 100 μg/ml of the αAC(71–88)Antp nanofiber; red plot, + 30 μ/g/ml of the neutral αAC nanofiber; cross plot, + 15μg/ml of the neutral αAC nanofiber. Each measurement was repeated three times. The plots are the averaged value (mean ± SD). (b) Time course of ThT fluorescence intensity of 2.2 mg/ml 3RMBD solutions incubated at 37°C. Black plot, 3RMBD alone; red plot, + 11 μg/ml of αAC(71–88) nanofiber; blue plot, + 44 μg/ml of the αAC(71–88)Antp nanofiber; cross plot, + 13 μg/ml of the neutral αAC nanofiber. Each measurement was repeated three times. The plots are the averaged value (mean ± SD). The concentration of ThT was 20 μM.

Next, the effect of theαAC(71–88)Antp nanofiber on the aggregation of Alzheimer pathogenic proteins was studied. The time trace of thioflavinT (ThT) fluorescence intensity showed that amyloid fibril formation of 100 μg/ml Aβ(1–42) was accelerated in the presence of 100 μg/ml αAC(71–88)Antp nanofiber (blue plot in Fig 2A), whereas αAC(71–88)Antp nanofiber itself had little effect on the ThT fluorescence at this concentration. (blue triangle plot in S3 Fig in S1 File) These results suggest that the major effect of the positively chargedαAC(71–88)Antp nanofiber is to accelerate the aggregation of anionic Aβ(1–42).

The time trace monitoring ThT fluorescence intensity shows that the aggregation of 2.2 mg/ml 3RMBD exhibits a slow and gradual increase in the presence of the negatively charged αAC(71–88) nanofiber (red plot in Fig 2B). By contrast, αAC(71–88)Antp nanofiber did not significantly stimulate the growth in aggregation of 3RMBD (blue plot in Fig 2B). These observations indicate that the positively charged nanofiber ofαAC(71–88)Antp only promotes the aggregation of negatively charged Aβ(1–42).

### Neutral αAC nanofiber prevents the aggregation of amyloid β

In order to suppress aggregation of Aβ (1–42) without promoting that of 3RMBD of tau, we designed an electrostatically neutralized αAC nanofiber. The αAC nanofibers composed of αAC(71–88) and αAC(71–88)Antp were prepared by the heat treatment of a solution mixtu e of these peptides at various molar ratios (αAC(71–88): αAC(71–88)Antp = 10:1, 4:1 and 2:1). TEM analysis showed that regular nanofibers were formed from these solution mixtures (Fig 1C). The average surface charge of the αAC nanofibers prepared from a 4:1 (αAC(71–88): αAC(71–88)Antp) solution mixture was close to zero (S4 Fig in S1 File).

We examined the effect of the neutral αAC nanofiber on amyloid fibril formation of Aβ and 3RMBD of tau by ThT assay. We confirmed that no ThT molecules bind to the neutral αAC nanofibers themselves in the concentration range less than 30 μg/ml (red plot in S3 Fig in S1 File). The time trace of ThT fluorescence intensity showed that amyloid fibril formation of 100 μg/ml Aβ(1–42) was suppressed by the neutral αAC nanofiber in the concentration range less than 30 μg/ml (red plots and cross plots in Fig 2A), in contrast that αAC(71–88)Antp promoted the Aβ(1–42) aggregation. A higher concentration of the neutral αAC nanofiber promoted Aβ(1–42) aggregation, probably because of self-association of the nanofiber itself. On the other hand, the neutral αAC nanofiber did not promote the aggregation of 3RMBD at the nanofiber concentration of 13 μg/mL(cross plot in Fig 2B).

**Fig 3:**
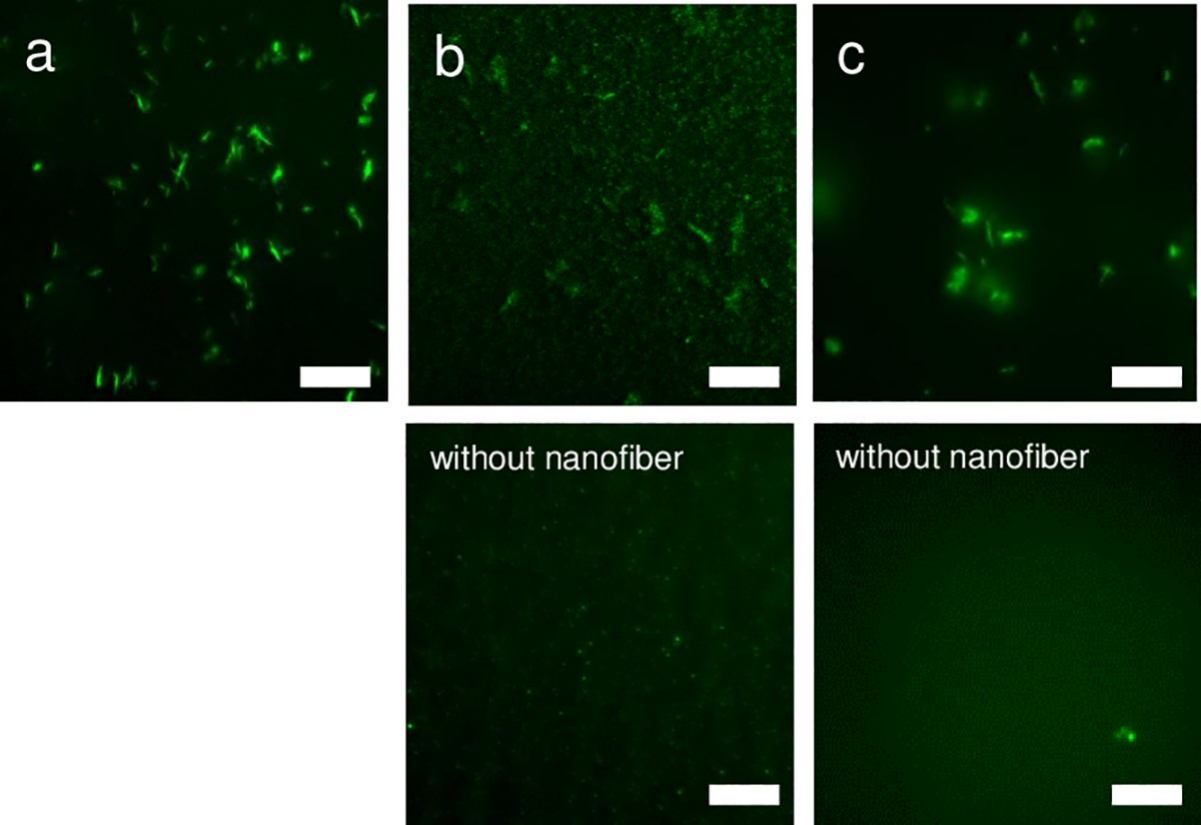
TIRFM analysis of protein binding to the neutral αAC nanofiber. (a) TIRFM image of the αAC nanofibers stained with 10 μM ThT excited by 457 nm light from an Ar^+^ laser. (b) TIRFM image of 100 nMHilyteFluor^TM^488 labeled Aβ (1–42) incubated with (upper panel) and without (lower panel) αAC nanofiber. Samples were excited by light at 488 nm from an Ar^+^ laser. (c) TIRFM image of 2 μM fluorescein labeled 3RMBD incubated with (upper panel) and without (lower panel) neutral αAC nanofiber. Samples were excited by light at 488 nm from an Ar^+^ laser. The concentration of the neutral αAC nanofiber in these figures was 60 μg/ml (peptide concentration; 23 μM). The exposure time was 200 ms in all experiments. The experiments were carried out repeatedly, and the reproducibility was confirmed. Each scale bar represents 10 μm.

We have proposed that the αAC nanofiber exhibits chaperone-like activity by trapping small protein aggregates on the nanofiber surface [29]. To monitor the binding of Aβ and tau protein on the neutral αAC nanofiber surface, we employed total internal reflection fluorescence microscopy (TIRFM), which can visualize the fluorescence image of the amyloid on a cover glass [35]. Fig 3A shows a TIRFM image of neutral αAC nanofibers on a cover glass probed with ThT excited by light at 457 nm. These results demonstrate that micrometer scale length fibrillar structures can be successfully observed by this methodology. The interaction between Aβ and the neutral αAC nanofiber was monitored using Hilyte Fluor^TM^488 labeled Aβ(1–42) excited by light at 488 nm. The TIRFM images of fluorescence-labeled Aβ(1–42) incubated with the neutral αAC nanofiber were similar to those stained by ThT (Fig 3B). When the neutral αAC nanofibers were observed in the absence of any fluorescence molecules by excitation at 457 nm or 488 nm, no images were obtained, showing that there was no autofluorescence under these con itions. Because ThT fluorescence probes were not used in the experiment (upper panel of Fig 3B), the observed fluorescence signals must be derived from Hilyte Fluor^TM^488 labeled Aβ(1–42). On the other hand, no nanofiber structures were observed in the TIRF images of fluorescence-labeled Aβ(1–42) without the neutral αAC nanofibers (lower panel of Fig 3B). Overall, these results suggest that Aβ(1–42) is attached to the neutral αAC nanofiber. The same result was obtained for the TIRFM image of the neutral αAC nanofiber probed by the fluorescent labeled 3RMBD (Fig 3C). These results demonstrate that the neutral αAC nanofiber suppresses the aggregation of Aβ and 3RMBD of tau by trapping them on their surface.

**Fig 4:**
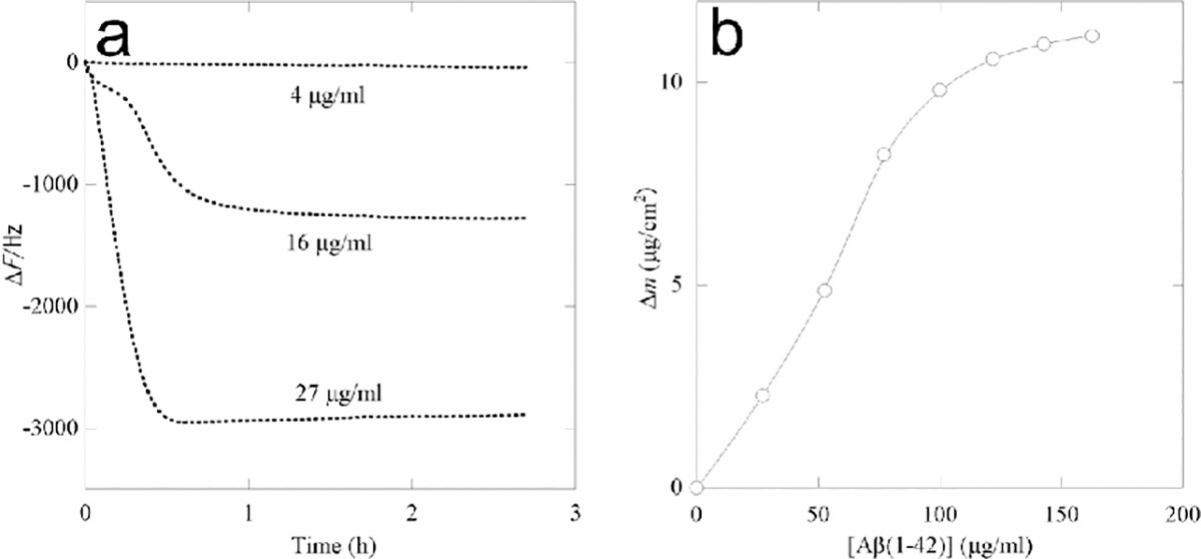
QCM analysis of protein binding to the neutral αAC nanofiber. (a) Time course of frequency changes (Δ*F*) of 27 MHz QCM responding to a binding of Aβ(1–42) to the neutral αAC nanofiber in the reaction vessel at 37°C with stirring. The nanofiber was immobilized on the sensor chip covered by carboxyl acid terminated self-assembled monolayers. (b) QCM analysis of the binding of Aβ(1–42) to immobilized neutral αAC nanofiber. Binding isotherm of Aβ(1–42) on the immobilized neutral αAC nanofiber. The amount of Aβ(1–42)bound to the neutral αAC nanofiber (Δ*m*) was calculated from Δ*F* monitored by a 27 MHz QCM at 37°C with stirring.

The interaction between Aβ (1–42) and the neutral αAC nanofiber was studied using a quartz crystal microbalan ce (QCM). The neutral αAC nanofiber was covalently immobilized onto a carboxylic acid terminated self-assembled monolayer prepared on the sensor chip. The amount of immobilized nanofiber was calculated to be 155 ng/cm^2^. The stock of Aβ(1–42) was diluted into a solution, and the time courses of Δ*F* upon addition of Aβ(1–42)solution were monitored (Fig 4A). While the change in Δ*F* was substantial at a Aβ(1–42) concentration of 4 μg/ml, a remarkable frequency decrease was monitored after the injection of higher concentrations of Aβ(1–42), which indicates its extensive binding to the neutral αAC nanofiber. The time course of the Δ*F* change is not exponential, indicating that this is not a simple binding mechanism. Presumably, aggregation of Aβ(1–42) and its binding to the neutral αAC nanofiber occur simultaneously in solution. The mass of Aβ(1–42) bound on the neutral αAC nanofiber at the different concentrations of Aβ(1–42) was estimated from each plateau value of Δ*F*. As shown in Fig 4B, the maximum mass of Aβ(1–42) bound on the neutral αAC nanofiber sensor chip was estimated to be >11 μg/cm^2^, which is much greater than the amount of neutral αAC nanofiber (155 ng/cm^2^). Therefore, a given quantity of the neutral αAC nanofiber can bind much larger amounts of Aβ(1–42), suggesting the multilayered adsorption of aggregates.

**Fig 5:**
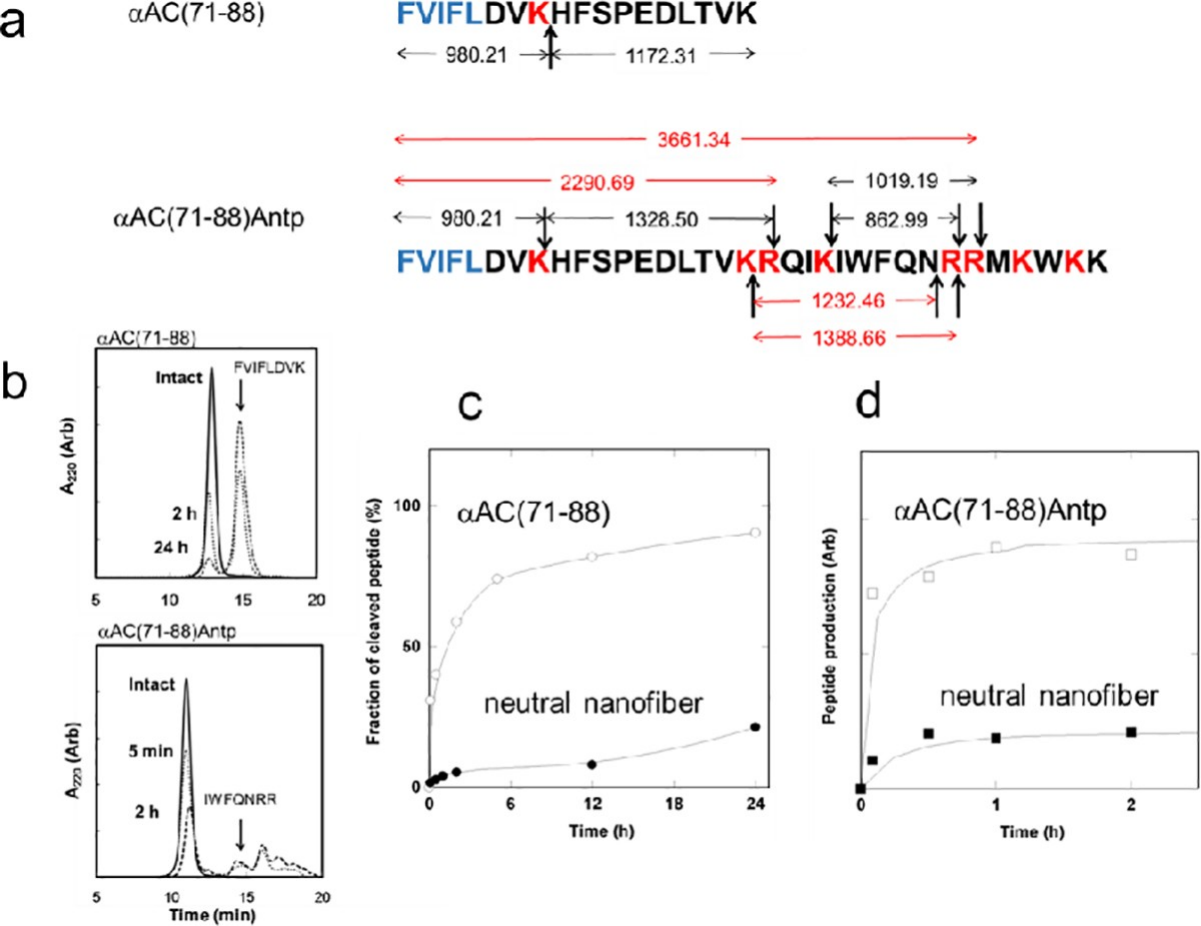
Proteolytic digestion of αAC peptide and the neutral αAC with trypsin. (a) Arrows indicate the location of the cleaved peptide bond after treatment with trypsin as determined by mass analysis of the peptide fragments. Except for the fragments colored in red, the same digestion products were detected in the MASS spectrum of neutral αAC nanofiber after treatment with trypsin. The protease trypsin mainly cleaves peptide bonds to the carboxyl side of lysine and arginine residues (highlighted in red). The FVIFL sequence, a core region of the nanofiber structure, is colored in blue. (b) Gel permeation chromatogram of αAC peptides treated with trypsin. The elution peak of the fragments of αAC(71–88) and αAC(71–88)Antp were detected by monitoring the absorbance at 220 nm. The same GPC profiles were found for the trypsin treated neutral αAC nanofibers except for the intact peptides, which were present in the form of nanofibers and were removed by centrifugation. (c) Time course for the proteolysis of αAC(71–88) and neutral αAC nanofiber. The degree of single cleavage of αAC(71–88) was determined by monitoring the intensity of the GPC peak for FVIFLDVK normalized against the intensity of the peak corresponding to intact peptide. (d) Time course for the proteolysis of αAC(71–88)Antp and neutral αAC nanofiber. The relative degree of proteolysis was estimated by analyzing the GPC peak intensity of the fragments of IWFQNRR (lower panel of Fig 5B).

The formation of a rigid nanofiber of αAC peptides is expected to reduce the susceptibility of the peptide to enzymatic degradation and assist in increasing the stability of the peptide *in vivo*. We used trypsin to examine the effect of nanofiber formation on the proteolytic digestion of αAC peptide, because trypsin has substrate specificity and mainly cleaves peptide bonds at the carboxyl sides of lysine and arginine residues, which allows for the analysis of fragmentation induced by enzymatic hydrolysis. Fig 5A shows the location of the cleaved peptide bonds after proteolytic digestion of the αAC peptides as determined from the molecular weight of the peptide fragments analyzed by MALDI TOF-MASS (S5 Fig in S1 File). The same pattern of fragmentation was produced by trypsin digestion of the neutral αAC nanofiber, except for four fragments of αAC(71–88)Antp (colored red in Fig 5A). The apparent rate of proteolysis of αAC peptide and the neutral αAC nanofiber was determined by GPC analysis of the trypsin treated samples (Fig 5B). The time course of the single cleavage of αAC(71–88) indicates that ca. 90% of the sample was digested after 24 h proteolysis (Fig 5C). By contrast, only ca. 20% of αAC(71–88)composing the neutral αAC nanofiber of was digested under the same experimental conditions. Similar results were obtained for the Antp sequence of αAC(71–88)Antp, which indicated (Fig 5D) that formation of nanofibers reduced the susceptibility of the peptide to proteolysis.

### *In vivo* function of the neutral αAC nanofibers of short length

We tested whether the nanofiber can be delivered efficiently into mouse brain *via* two administration pathways, intravenous or intranasal administration [33, 36]. At first, we investigated the distribution of the fluorescent-labeled neutral αAC nanofibers in the brain after intravenous administration of the nanofiber. However, only a small fraction of the nanofibers was delivered to the brain. We believe that the length of neutral αAC nanofibers affected their efficient delivery to the brain. Then, the neutral αAC nanofiber with a shorter length, which would be suitable for delivery into the brain parenchyma, was prepared by a modified preparation procedure using low temperature conditions. The nanofibers were prepared from a solution mixture of different molar ratios of peptides (αAC(71–88): αAC(71–88)Antp = 4:1, 2:1, 1:1 and 1:2) by the freeze-thaw method. Electrostatically neutralized αAC nanofibers with a length of less than 100 nm were successfully prepared from a 2:1 solution (S6 Fig in S1 File).

**Fig 6:**
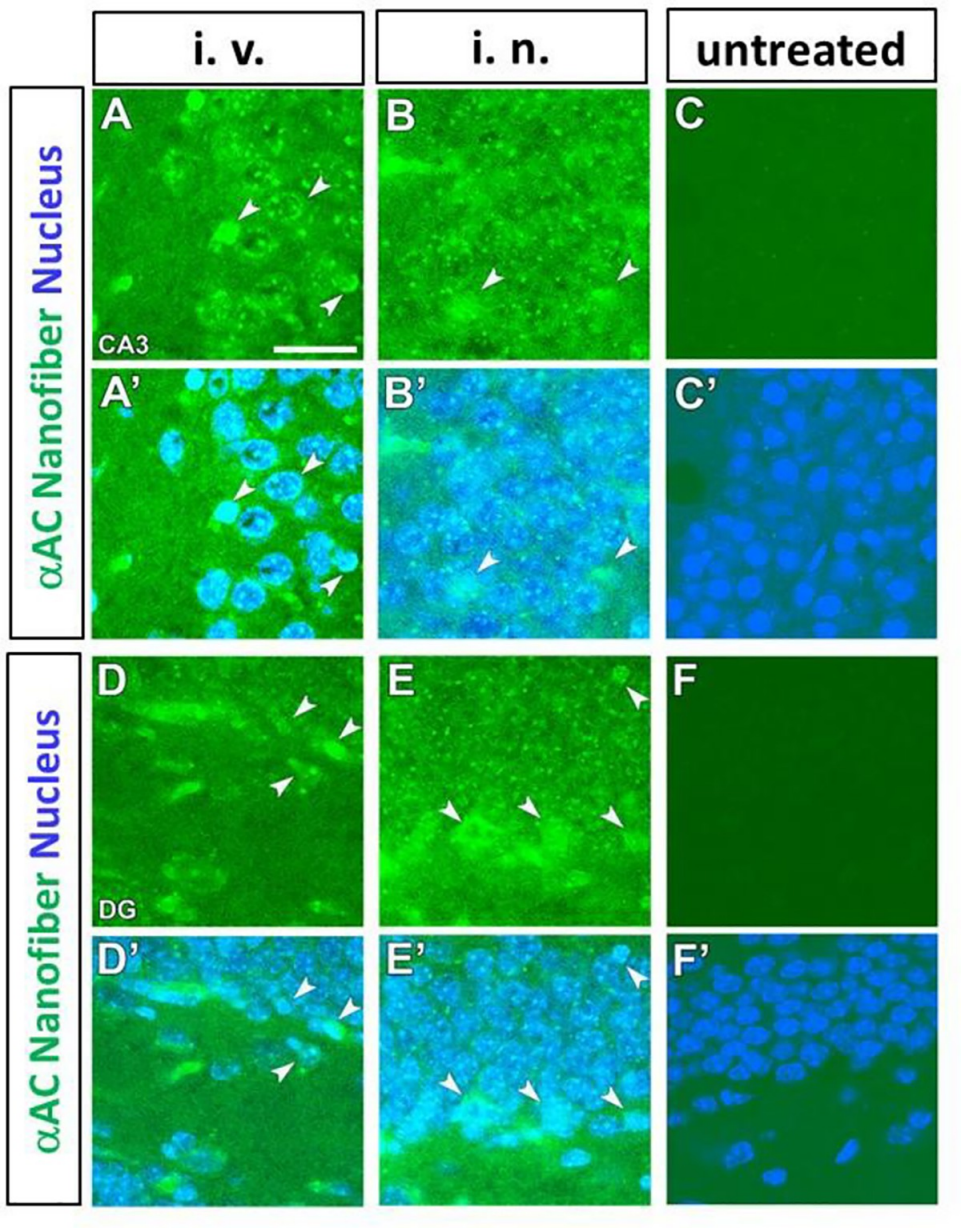
Confocal microscopic images showing the presence pf prominent fluorescence at the cornu ammonis (CA3) (A-C, A’-C’) and dentate gyrus (DG) (D-F, D’-F’) of mouse hippocampus after intravenous (i. v.) and intranasal (i. n.) administration of fluorescein-labeledαAC nanofibers. The αAC nanofibers are colored green. The nuclei of neurons stained with 4',6-diamidino-2-phenylindole (DAPI) are colored blue. Arrowheads indicate that the nanofibers are incorporated into cells. All experiments were performed at least three times. Scale bar represents 10 μm.

A 100 μl aliquot of fluorescently-labeled neutral αAC nanofiber (600 μg/ml) was intravenously injected into mice and their brains were collected 30 min after injection. The collection time was determined, based on the literature reporting pharmacokinetic analysis of proteins co-administered with cell-penetrating peptides via the intranasal pathway [34]. Fig 6A–6D show confocal microscopic images of the CA3 and dentate gyrus of the hippocampus, respectively. These images indicate that the fluorescence of nanofiber (green) is detected at the pyramidal cells and granule cells after the intravenous administration. There are some cells where the nanofibers were located in the nucleus of neurons stained with 4’6-diamidino-2-phenylidiole (blue). Images recorded under the same condition show no fluorescence signals without the administration of the neutral αAC nanofiber. (Fig 6C–6F). In addition, when the neutral αAC nanofibers were administered via intranasal pathway, successful delivery into the hippocampus also was observed (Fig 6B–6E).

**Fig 7:**
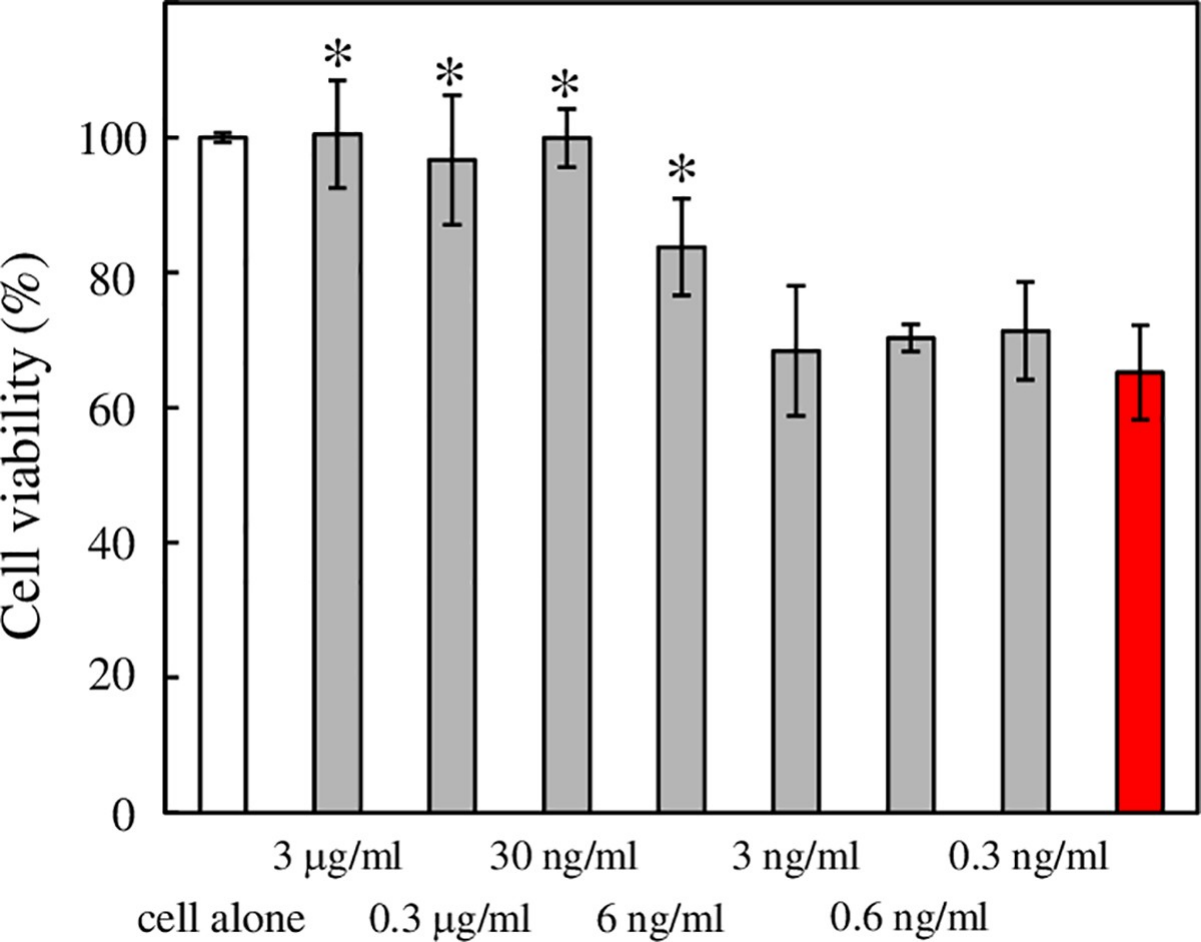
Ability of the neutral αAC nanofibers to protect PC12 cells from the cytotoxicity of Aβ(1–42). Cells were treated with 5 μg/ml (1 μM) Aβ(1–42) for 24 h before determining their viability using the lactate dehydrogenase method. Red bar, cell viability in the absence of the neutral αAC nanofiber; grey bars, cell viability in the presence of 0.3 ng/ml—3 μg/ml (peptide concentration; ca. 0.1 nM -1 μM) of αAC nanofiber; white bar, cell viability in the absence of αAC nanofiber and Aβ(1–42). The results are expressed as percentages of the control value (mean +- SD). *, p < 0.01 by Dunnett's test when compared with cell viability in the absence of the neutral αAC nanofiber.

The effectiveness of the short length neutral αAC nanofibers to treatment AD was evaluated by analyzing the suppression of the cytotoxicity of Aβ (1–42) using PC12 cells as a model. The viability of PC12 cells in the presence of various concentrations of neutral αAC nanofiber was monitored by the lactate dehydrogenase (LDH) assay. First, the cytotoxicity of the neutral αAC nanofibers to PC12 cells was investigated. The nanofibers did not exhibit detrimental effects on cells during co-incubation for 24 h at concentrations below 0.15 mg/mL (S7 Fig in S1 File). Fig 7 shows the cell viability of PC12 cells after 24 h incubation with 5 μg/ml (ca. 1 μM) Aβ (1–42). In the absence of neutralαAC nanofiber, the incubation of Aβ (1–42) with PC12 cells resulted in ca. 35% cell death over the same time period. In the presence of the neutral αAC nanofiber, viability of PC12 cells was increased as the concentration of the neutral αAC nanofiber increased from 3 ng/ml to 3 μg/ml (peptide concentration; ca. 1 nM—1 μM). Indeed, the cytotoxicity of 5 μg/ml Aβ (1–42) was almost completely suppressed in the presence of more than 30 ng/ml (peptide concentration; ca. 10 nM) of the neutralαAC nanofiber.

### Effect of the short length neutral αAC nanofiber treatment on transgenic AD model mice

Next, we examined the beneficial effects of the short length neutral αAC nanofiber on cognitive deficits using APPswe/PS1dE9 transgenic mice [37]. We mainly focused on the efficacy of the intranasal injected-nanofibers, because intranasal administration is non-invasive treatment, which is advantageous in practical [38,39].

**Fig 8:**
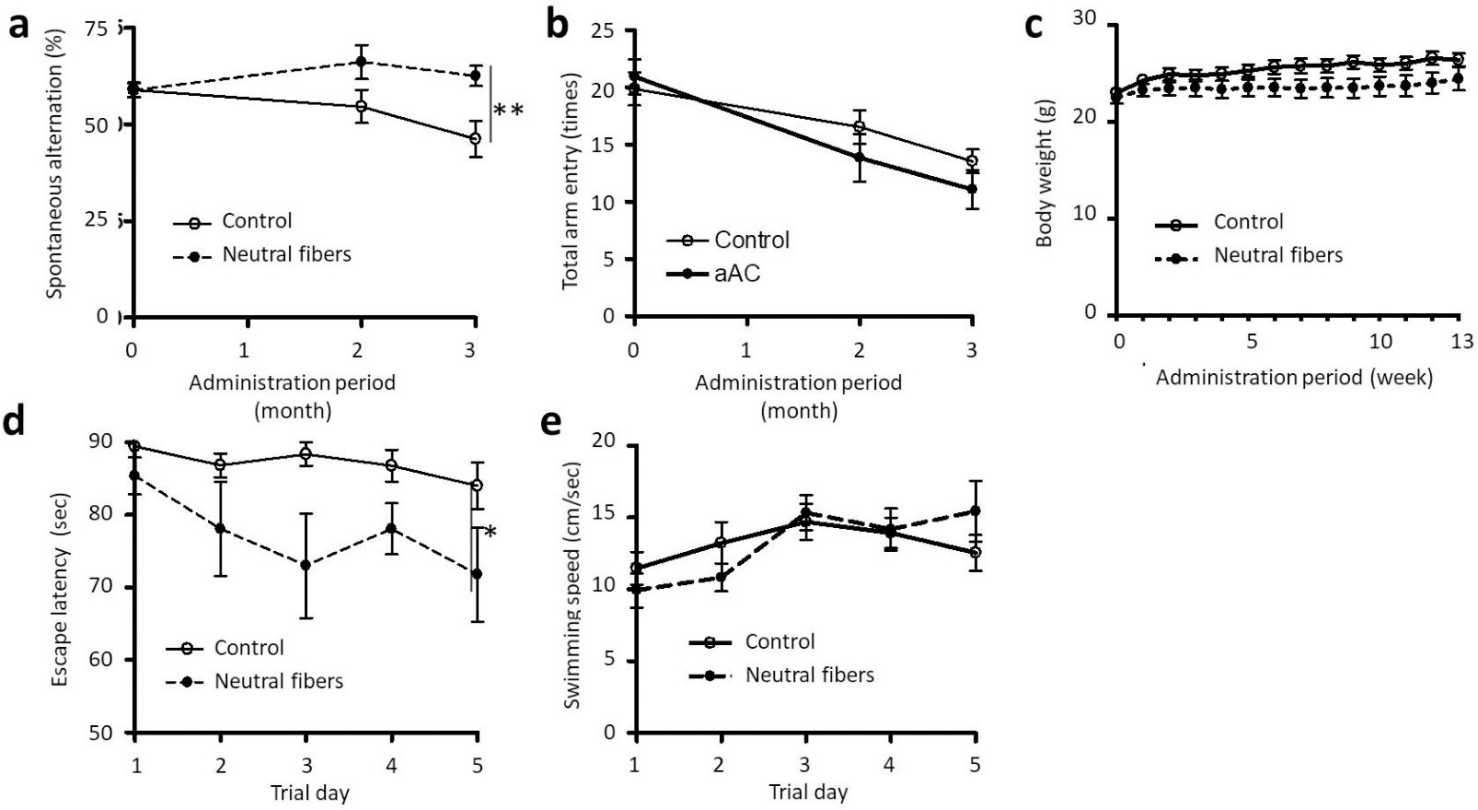
Beneficial effects of αAC nanofiber on cognitive dysfunction in APPswe/PS1dE9 transgenic AD model mice. (a) Percentage of spontaneous alternation behavior in the Y-maze test. **P = 0.005, two-way analysis of variance (ANOVA). The neutral αAC nanofibers were intranasal administered to female mice at ca. 1.5 mg/kg twice weekly for 8 weeks. The open and closed circle plots indicate the vehicle (n = 13) and neutral αAC nanofiber group (n = 13), respectively. (b) The change in total number of arm entry in the Y-maze test. (c) The change in body weight of the AD model mice. (d) Escape latency in the Morris water maze test. **P = 0.007, two-way ANOVA. Mean ± SEM, n = 13. (e) Swimming speed of the AD model mice in the Morris water maze test.

The neutral αAC nanofiber was intranasally administered to 3-month-old female APPswe/PS1dE9 transgenic mice. During the treatment, the spatial working memory of the mice was evaluated using the Y-maze test (Fig 8A and 8B). At the start of treatment, the percentage of spontaneous alternations in each group was about 60% (59.8 ± 1.9% in the control group, 59.8 ± 1.7% in the neutral αAC nanofiber-treated group). In the control group, the percentage of spontaneous alternations reduced with age to 46.2 ± 4.6% in the 13th week of treatment. In contrast, in the nanofiber-treated group, the percentages of spontaneous alternations remained the same as at the start of treatment (61.4 ± 2.7%). There was a significant difference between these groups (Fig 8A). There was no significant difference in the total arm entry between the groups (Fig 8B). The average weight of each group during the treatment is shown in Fig 8C. Although some weight loss was observed in the nanofiber-treated group, there was no significant difference between the groups. In addition, no abnormalities were observed due to the nanofiber. These results suggest that neutral αAC nanofiber is very safe.

Furthermore, the spatial learning of the mice was assessed using the Morris water maze test at the 13th week of treatment (Fig 8D and 8E) [40]. In the control group, the escape latency did not alter during the trial (83.9 ± 3.2 s on the fifth day). In contrast, in the neutral αAC nanofiber-treated group, the escape latency decreased (71.7 ± 6.5 s on the fifth day) and there was a significant difference between these groups (Fig 8D). There was a tendency for increased swimming speed in the treatment group; however, no significant differences were observed (Fig 8E). These results clearly show that intranasal injected- nanofiber improved the cognitive function of an Alzheimer transgenic mouse model.

**Fig 9:**
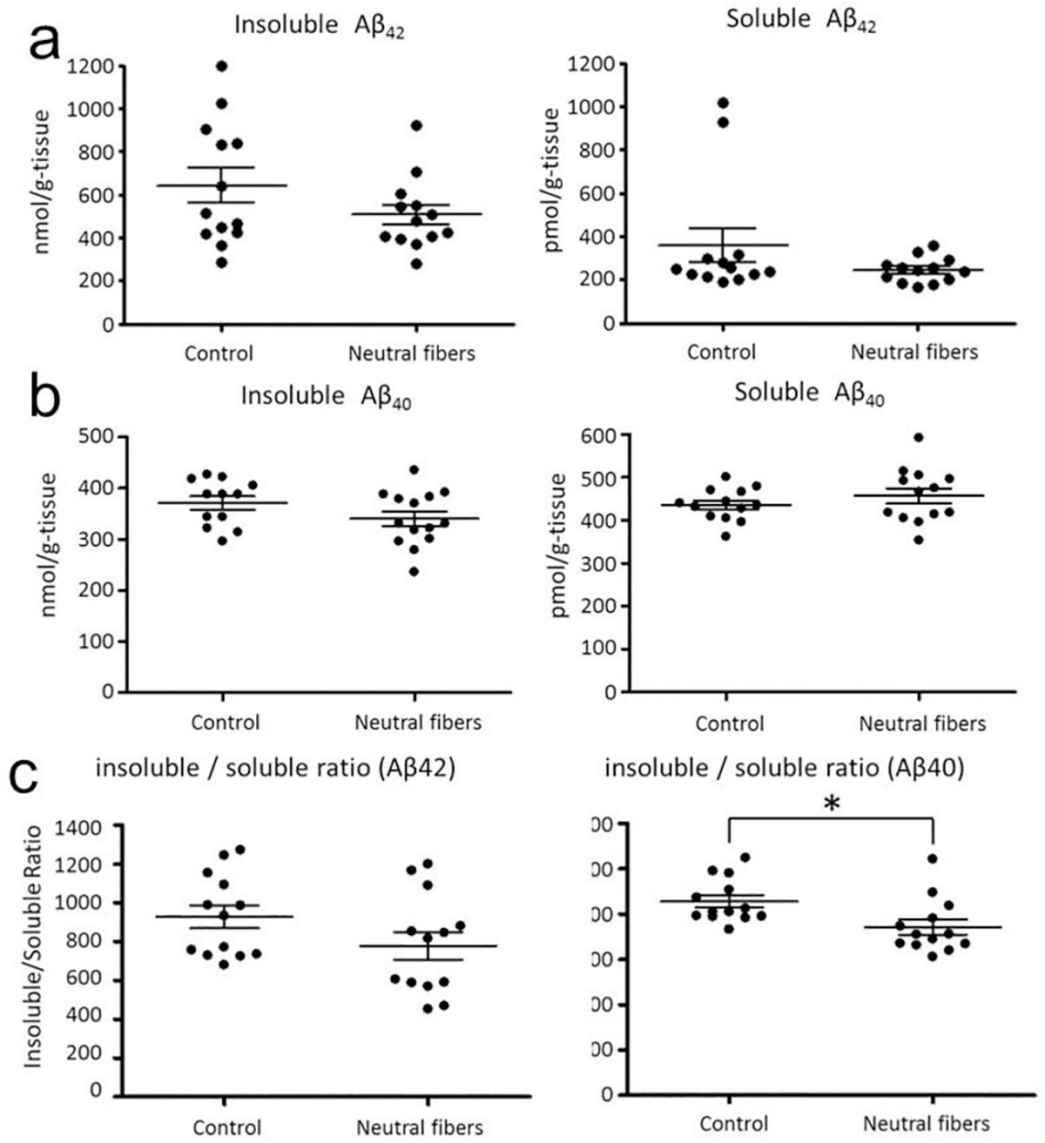
The amount of insoluble and soluble Aβ in the brains of APPswe/PS1dE9 transgenic mice. Mean ± SEM, n = 13. *P = 0.014, Dunnett's test. (a) Aβ(1–42), (b) Aβ(1–40), (c) the ratio of soluble / insoluble Aβ(1–40).

The amount of Aβ in the brain after nanofiber treatment was measured using ELISA (Fig 9A, 9B and 9C). The average level of insoluble Aβ(1–42) decreased of 21% in the neutral αAC nanofiber-treated group and that of insoluble Aβ(1–40) decreased of 10% in the neutral αAC nanofiber-treated group, but resulted in no significant difference. Similarly, the amounts of soluble Aβ(1–42) and Aβ(1–40) were not significantly different between the groups. However, the ratio of insoluble/soluble Aβ(1–40) was significantly different between the groups.

In addition, we obtained the preliminary findings that intravenously injected-nanofibers also improve the performance of APPswe/PS1dE9 transgenic mice. NeutralαAC nanofiber was given intravenously over an 8 week period to 5-month old mice. As shown in S8 Fig in S1 File, the percentage of spontaneous alternation behavior at the eighth week was 76.0 ± 1.0% for the treatment group and only 53.0 ± 7.0% for the vehicle group. The difference between these two groups is sufficiently significant (p = 0.04). After the treatment, brain sections of mice were prepared and Aβ plaques were immune stained with anti-Aβ antibody 6E10 (S9 Fig in S1 File). There was a tendency for decreased area (S9a Fig in S1 File) and abundance of Aβ plaques (S9b Fig in S1 File) in the neutralαAC nanofiber-treated group, compared with the vehicle group. Interestingly, the average size of Aβ plaques tended to increase in the treatment group (S9c Fig in S1 File). These results supported the notion that neutral AC nanofibers localized in brain exhibits preferable efficacy on cognitive deficits.

## Discussion

In this study, we developed electrostatically neutral αAC nanofibers for AD treatment. The design concept is based on our previous findings, which are described below. We found that the nanofibers comprising αAC(71–88) peptides can inhibit the aggregation of alcohol dehydrogenase. In contrast, other studies showed that the protein aggregation was facilitated by the peptide nanofibers [41,42]. To explain this discrepancy, the previous study investigated the effect of peptide nanofibers on the aggregation of various proteins. We showed that the apparent surface charge of the nanofiber is the key factor determining the suppression or promotion of protein aggregation [29]. When the surface charge of nanofibers is equivalent to that of substrate proteins, binding of the proteins to the nanofiber surface, possibly via hydrophobic interactions, induces reduction in the concentration of free proteins, suppressing the aggregation of the substrate proteins. Conversely, when the surface charge of nanofibers is opposite to that of substrate proteins, binding of the proteins to the nanofiber surface via electrostatic interactions leads to the cancelation of the surface charge, promoting an irreversible aggregation of the substrate protein. This is a disadvantage of using the peptide nanofibers for AD-treatment, because not only the aggregation of Aβ proteins, but also that of tau proteins should be targeted: the charged nanofibers suppress the aggregation of one protein, but promote that of the other because Aβ molecules (pI = 5.5) and 3RMBD of tau (pI = 9.6) [30, 31] have opposite net charges at neutral pH. In fact, our previous study indicated that the aggregation of the anionic Aβ (1–40) was suppressed by the negatively charged nanofiber of αAC(71–88), while that of the cationic fragment of 3RMBD of tau protein was promoted. Thus, we believe that neutralization can solve this charge dilemma. In the present study, the electrostatically neutral αAC nanofibers were prepared by optimizing the mixing ratio of the anionic αAC(71–88) peptides and the cationic variants αAC(71–88)Antp peptides (Fig 1C; S4 Fig in S1 File). Fig 1A shows proposed structures of the neutral peptide nanofibers, estimated on the basis of our previous studies [43, 44].

The neutralαAC nanofibers administered to the mouse, either via the intranasal pathway or the intravenous pathway, was confirmed to be distributed in the brain 30 min after administration (Fig 6). Antp sequence may enhance the uptake of nanofibers by nasal epithelial cells (neuronal or supporting cells), facilitating the transport of the nanofibers to the brain [34]. The intravenously injected-nanofibers may be delivered into the brain via mechanisms proposed for the cationic cell penetration peptides, such as direct penetration through the membrane [45] or adsorptive-mediated transcytosis [46]. Alternatively, the amphiphilic nature of the peptide nanofiber could potentially promote its transport across the BBB [47]. Our results indicate that the neutral αAC nanofibers were incorporated into the hippocampus, indicating that the nanofibers could penetrate the BBB and be successfully delivered into brain parenchymal cells. In addition, the susceptibility of the αAC(71–88) and αAC(71–88)Antp peptides to enzymatic hydrolysis was reduced by nanofiber formation (Fig 5). This property contributes to the stability of the neutralαAC nanofibers *in vivo*.

The neutral αAC nanofibers suppressed aggregation of Aβ (1–42), without promoting the aggregation of 3RMBDof tau (Fig 2) and cytotoxicity of Aβ (1–42) against PC12 cells (Fig 7). Several brain-permeable low molecular weight compounds that suppress the aggregation of amyloidβ have been proposed as therapeutic drugs for AD [48–55]; unfortunately, the affinity of these compounds for Aβ tends to be low. For example, >100 nM of curcumin is required to inhibit the cytotoxicity of 100 nM of Aβ (1–42) [49]. By contrast, our data shows that a much lower concentration of nanofiber (peptide molar concentration of ca. 10 nM) can inhibit the cytotoxicity of ca. 1 μM Aβ (1–42) (Fig 7). TIRF images of the neutral αAC nanofibers co-incubated with fluorescence-labeled Aβ (1–42) revealed that the Aβ (1–42) molecules adsorb on the surface of the neutralαAC nanofibers (Fig 3). In addition, the adsorption of Aβ (1–42) was quantitively shown by QCM study, indicating that the neutral αAC nanofiber can bind a much greater amount of Aβ (1–42) than itself (Fig 4). Thus, the neutral αAC nanofiber appears to trap large quantities of Aβ (1–42) aggregate, thereby suppressing its aggregation and cytotoxicity. We propose that a large hydrophobic surface present on the nanofiber can trap Aβ (1–42) more efficiently than low molecular weight compounds.

The cognitive functions of AD-model mouse were improved by either intranasal or intravenous administration of the neutral αAC nanofibers (Fig 8; S8 Fig in S1 File). Other studies have demonstrated the ability of peptide nanofibers to effectively treat various diseases [9, 10, 56–58]. A peptide nanofiber with the IKVAV sequence, the neuroactive pentapeptide epitope from laminin, displays therapeutic effects in a transgenic mouse model of AD by direct injection into the hippocampus [56]. However, the direct injection of peptide nanofiber into the brain of patients with AD is impractical. Importantly, we were able to demonstrate that even intranasally delivered neutral αAC nanofibers improve the cognitive function of an Alzheimer transgenic mouse model. Given that intranasal delivery offers a painless and convenient method of self-administration by patients, we believe our neutral αAC nanofibers will be useful for the practical treatment of AD in the near future.

However, the mechanism on the cognitive function improvement of AD-model mouse by nanofiber treatment has been debatable. Intranasal administration of nanofibers resulted in a negligible decrease in the amount of Aβ(1–40) and Aβ(1–42), soluble or insoluble, in the brain (Fig 9). However, the ratio of insoluble/soluble Aβ(1–40) in the nanofiber-treated group was slightly reduced compared to that in the control group, suggesting that Aβ aggregation is inhibited in neutra αAC nanofiber-treated mice, rather than Aβ production or clearance. This is one possible reason for the improvement of cognitive function by nanofiber treatment. However, because the difference in the ratio between groups was less pronounced, other possibilities cannot be rejected. Recently, soluble oligomers of Aβ aggregates are regarded as the main cause of neurotoxicity [6]. Nanofibers trap oligomer species of Aβand prevent them from interacting with nerve cells, causing harm; this is a possible role of nanofibers in the improvement of AD-mouse cognitive function. Moreover, the observation of immunostained brain sections revealed that the size of Aβ plaques in the brain of the mice treated with nanofibers was larger than those of the control group, indicating that the morphology of Aβ plaque was influenced by nanofibers. (S9 Fig in S1 File). In previous studies, some kinds of flavone, kaempferol-3-O-rhamnoside and taiwniaflavone, directly bound to the unfolded Aβ molecules and promoted formation of nontoxic structures, which were significantly different from Aβ fibril structures formed in the absence of the compounds [59, 60]. Similarly, the neutral αAC nanofibers possibly affect the assembly of Aβ molecules to produce less toxic aggregates, leading to the recovery of the AD-mouse cognitive function. This should be further explored in future investigations. In addition. to clarify the mechanism, investigation of pathological states of brain, by immunostaining of inflammation markers is also an important future study to consider.

In the present study, we proposed a novel design concept of nanofibers for AD-treatment. Our results from *in vitro* experiments imply that the charge neutralization is beneficial for preventing the aggregation of Aβ without promoting the aggregation of 3RMBD of tau and to suppress Aβ cytotoxicity against PC12 cells. Behavior tests using AD-model mice also shows that the administration of neutral αAC nanofibers can improve their cognitive function, although the mechanism is unknown. However, whether the nanofiber design is optimal for *in vivo* application or not has yet to be clarified, because the surface charge of the nanofibers may change by binding the biomolecules *in vivo*. To verify the design concept, the effect of nanofiber charge on their ability to improve the cognitive function of AD-model mice using negatively or positively charged nanofibersshould be investigated.

## Conclusions

In conclusion, we have prepared an electrostatically neutral αAC nanofiber from a solution of αAC(71–88) and αAC(71–88)Antp. The neutral αAC nanofiber suppressed the aggregation of Aβ by trapping the proteins on the nanofiber surface. The short length nanofibers prepared by the freeze-thaw procedure were brain-permeable, and improved the cognitive function of an Alzheimer transgenic mouse model. Therefore, the neutral αAC nanofiber is expected to be a promising therapeutic peptide nanofiber for the treatment of AD.

## Materials and methods

### Peptides and proteins

Aβ (1–42) were obtained from Peptide Institute (Osaka, Japan) and HilyteFluor^TM^488 labeled Aβ (1–42) was purchased from ANASPEC (Fremont, CA). Other peptides used in this study were synthesized by Gene script (Piscataway, NJ). Peptides were purified by reverse phase HPLC using a C18 column with a water-acetonitrile gradient. The expression vector for three-repeat microtubule-binding domain (3RMBD) of human brain tau protein was kindly provided by Prof. T. Ishida (Osaka University of Pharmaceutical Science) [30]. Gene expression and purification of His-tagged 3RMBD were performed as described previously [61], except that 8 M urea was included in the lysis buffer. In brief, 3RMBD overexpressed in *E*. *coli* was first purified using Ni-NTA resin. In this process, 8 M urea was gradually removed from the buffer. The dimeric protein, which is a building block of fibrillar aggregates [31], was separated by gel filtration on a Superdex 75 column and used for the aggregation study. Unless stated otherwise, 50 mM sodium phosphate (pH 7.5) with 100 mM NaCl was used as the solvent for all subsequent experiments.

### Fluorescent labeling of peptides and proteins

Fluorescein labeled AC(71–88) was prepared by modification of FVIFLDVKHFSPEDLTVKC (αAC(71–88)Cys) with 5'-iodoacetamidofluorescein (5-IAF). 100 μM αAC(71–88)Cys and 100 μM 5-IAF was incubated in an aqueous solution adjusted to pH 7.5, and unreacted 5-IAF was eliminated using a Centri spin column (Princeton Separations, Adelphia, NJ). The desired modification was confirmed by mass spectrometry (ESI-TOF-MASS using a Bruker Daltonics microTOF_LC_; Bruker Daltonics, Billerica, MA). Fluorescein labeled 3RMBD was prepared by modification of the N-terminus of the peptide with fluorescein isothiocyanate (FITC). 120 μM 3RMBD and 2.4 mM FITC was incubated in aqueous solution adjusted to pH 8.0, and unreacted FITC was subsequently removed using a NAP10 column.

### Preparation of the peptide nanofiber

The αAC peptides were dissolved in 5 mM phosphate, 100 mM NaCl, pH 7.5, 10% HFIP, and the solution was incubated at 60°C for 24 h to prepare the αAC nanofibers. For the freeze-thaw procedure, the solution was incubated in liquid nitrogen for 1 min. The solution was then centrifuged at 10,000 *g* for 15 min to separate the free peptide. The fluorescence-labeled peptide nanofibers were prepared as below. Non-fluorescence labeled αAC(71–88) peptide and αAC(71–88)Antp peptide were dissolved in 5 mM phosphate, and 100 mM NaCl, pH 7.5, at concentrations of 162 μM and 90 μM, respectively. Fluorescein labeled αAC(71–88) peptides were dissolved in HFIP at a concentration of 180 μM. The two solutions were then mixed at a volume ratio 10: 1 (the former: the later). The mixed solution was incubated in liquid nitrogen for 1 min, and then centrifuged at 10,000 *g* for 15 min.

### CD spectroscopy

The peptide secondary structure was monitored by CD spectroscopic measurements using a Jasco J-720 instrument (Jasco Corp., Tokyo, Japan). An optical cell with a 1 mm pathlength was used. Far-UV spectra of a 200 μg/ml peptide solution were measured at a scan speed of 20 nm/min at 25°C. 50 mM sodium phosphate (pH 7.5) with 100 mM NaCl was used as the solvent.

### Transmission Electron Microscopy (TEM)

TEM images of the αAC nanofibers were acquired with a JEM-1200EX II instrument (JEOL, Tokyo, Japan) using an acceleration voltage of 85 keV. The samples were negatively stained with 1.5–2.0% phosphotungstate adjusted to pH 7.5 using sodium hydroxide.

### Fluorescence measurements

Fluorescence spectra of ANS were obtained using a Shimadzu RF2000 spectrofluorimeter (Shimadzu, Tokyo, Japan). Amyloid fibril formation of Aβ (1–42) (100 μg/ml) was monitored by measuring the fluorescence intensity of 20 μM thioflavinT (ThT) in 50 mM phosphate pH 7.5 100 mM NaCl. The fluorescence measurements were carried out using a Genios plate reader (TECAN, Männedorf, Switzerland) with an excitation wavelength of 450 nm and emission wavelength of 485 nm in a polystyrene 96-microwell plate. The measurements were performed in triplicate. The lyophilized sample of Aβ (1–42) was first dissolved in 0.1% ammonia solution to prepare the stock solution. The stock solution of Aβ (1–42) was centrifuged at 53,000 rpm (> 100,000 *g*) using a Himac CS120GX ultracentrifuge (Hitachi Koki Co., Ltd, Tokyo, Japan) to eliminate the preformed aggregates, and diluted into the buffer. Fibril formation of 300 μg/ml tau 3RMBD was also monitored under the same conditions.

### Electrophoretic mobility measurements

The electrophoretic mobility of the αAC nanofiber was measured by laser microscopy using a Model 502 microscope (Nihon Rufut Co, Ltd., Tokyo, Japan) at 25°C. The motion of individual amyloid fibrils was visualized using light scattering images generated from a He-Ne laser (630 nm). Digital images of particles in motion captured with a CCD camera were transferred to a PC in order to calculate their electrophoretic mobility. The zeta potential was calculated using the Smoluchowski equation:

$$\mu_{e}=\frac{\varepsilon \zeta }{\eta }$$

where μ_e_ is the electrophoretic mobility, ε is the dielectric constant, ζ is the zeta potential and η is the viscosity.

### Total Internal Reflection Fluorescence Microscopy (TIRFM)

αAC nanofibers, Hilyte Fluor^TM^488 labeled Aβ (1–42) and fluorescein labeled 3RMBD on a cover glass were visualized using total internal reflection fluorescence microscopy (TE2000-TIRF2; Nikon, Tokyo, Japan). An Ar^+^ laser (IMA1010 40ALS; CVI Melles Griot, Albuquerque, NM) was used for excitation (ThT; 457 nm, Hilyte Fluor^TM^488 and FITC; 488 nm). The cover glass was treated with a UV ozone cleaner (model UV253E; Filgen Inc., Nagoya, Japan). The fluorescence emission was collected using an oil-immersion microscope objective (1.49 NA, 100 x, CF1 Apo TIRF; Nikon). Fluorescent images were filtered with a band-pass filter (for ThT, D490/20 m; Chroma Technology, Bellows Falls, VT; for FITC, GFP(R)-Band Pass 510–560 nm; Nikon) and visualized with an electron multiplier CCD camera (ImagEM C9100-13; Hamamatsu Photonics, Shizuoka, Japan).

### Quartz-crystal microbalance (QCM)

AFFINIX Q4 was used as a 27 MHz QCM instrument (Initium Co. Ltd, Tokyo, Japan: http://www.initium2000.com) having four 500 μL cells equipped with a QCM plate (8.7 mm diameter quartz plate; 4.9 mm^2^ gold electrode) at the bottom of each cell, a stirring bar and a temperature control system. The instrument was calibrated with a change frequency of 1 Hz in response to a mass change of 0.62 ng/cm^2^ on the electrode (i.e., corresponding to 1 Hz frequency decrease per 30 pg mass increase).

The αAC nanofiber was immobilized on the sensor chip using an immobilization kit coupling for AFFINIX (Initium Co. Ltd., Tokyo, Japan). The amino group of the peptide nanofiber was covalently linked to the carboxylic acid terminus of self-assembled monolayers prepared on the sensor chip by (3-dimethylaminopropyl)-3-ethylcarbodiimide (EDC)-*N*-hydroxysuccinimide (NHS) activation. The amount of immobilized neutral αAC nanofiber on the sensor chip was 155 ng/cm^2^. The stock solution Aβ (1–42) was diluted into buffer (50 mM phosphate pH 7.5, 100 mM NaCl) in the reaction vessel at 37°C with stirring. Binding of Aβ (1–42) to the αAC nanofiber was monitored by measuring the frequency change (Δ*F*, Hz) obtained according to the manufacture's protocol using the Sauerbrey equation:

$$\Delta F=-\frac{2{F_{0}}^{2}}{A\sqrt{\rho_{q}\mu_{q}}}\Delta m$$

where *F*_0_ is the fundamental frequency of the QCM (27 x 10^6^ Hz), Δ*m* is the mass change (g), *A* is the electrode area (4.9 mm^2^), ρ_q_ is the density of quartz (2.65 g cm^-3^), and μ _q_ is the shear modulus of quartz (2.95 x 10^11^ dyn cm^-2^).

### Atomic force microscopy (AFM)

A 20 μl aliquot of a 20-fold diluted αAC nanofiber solution was deposited onto freshly cleaved mica and dried immediately using a stream of nitrogen gas. The samples were imaged with a Nanoscope IIIa (Veeco Instruments, Santa Barbara, CA) in tapping mode, and the cantilever was set vibrating in the z direction at a resonance frequency of 290 kHz. The images were taken in air under ambient conditions using silicon tips.

### Proteolytic degradation

The αAC peptides or the neutralαAC nanofiber (200 μM) were incubated with trypsin (12 μM, type XIII from bovine pancreas) in 5 mM phosphate buffer containing 100 mM NaCl (pH 7.5) at 37°C. The molecular mass of the digested peptides were analyzed with MALDI-TOF mass spectroscopy using a BrukerAutoflex Speed instrument. The proteolyzed sample of αAC nanofibers was centrifuged (10,000*g* for 5 min) to separate the peptide fragments from the nanofibers. The apparent rate of proteolysis of αAC(71–88) was estimated from gel permeation chromatography analysis using a Superdex^TM^peptide 10/300 GL column (GE Healthcare, Piscataway, NJ). Water including 30% acetonitrile and 0.1% TFA was used as the elution solvent.

### Cytotoxicity assay

The Aβ(1–42) sample for the cytotoxicity test was prepared as follow;[62] Aβ(1–42) was dissolved in HFIP at 1 mg/ml. The solution was lyophilized to yield the powder. The obtained powder was dissolved in pure water and the solution was sonicated for 15 min. The aqueous solution was provided for the cytotoxicity assay. Rat PC12 cells (10,000 cells per well) was purchased from the RIKEN BRC (Tsukuba, Japan). were plated on collagen I-coated dishes in DMEM medium containing 5% heat-inactivated horse serum and 5% heat-inactivated fetal bovine serum and then incubated in a humidified 5% CO_2_ atmosphere at 37°C. The cytotoxicity induced by exposure to 5 μg/ml (ca. 1 μM) Aβ(1–42) was studied using PC12 cells. A sample of neutral αAC nanofibers (0.3 ng—3 μg /ml) was added to the culture after replacement of the medium with serum-free DMEM before treating the cells with Aβ(1–42). After 24 hour incubation, a LDH release assay was performed to evaluate the chaperone-like activity of the neutral αAC nanofiber against the cell cytotoxicity of Aβ(1–42). A commercial assay kit for LDH activity (Wako Pure Chemical Industries, Ltd. Osaka, Japan) was used. Assays were carried out by measuring the absorption at 560 nm using a Multiskan JX instrument (Thermo Labsystems, Helsinki, Finland). All measurements were performed in quadruplicate. The viability of cells treated with 0.5% Triton X-100 was assumed to be 0%. Control samples containing only the neutral αAC nanofiber in the same concentration range showed no detectable cytotoxicity. The results of the cell viability assays were analyzed by Dunnett's test. The software GraphPad Prism was used to perform these statistical analyses, and p < 0.05 was considered significant.

### Fluorescence confocal microscopy of mouse brain

Adult C57BL/6J mice of 10-14-weeks of age were used in the present study. The animals were housed two per cage in a colony room with a 12 h light (6:00–18:00) /12 h dark (18:00–6:00) cycle and given *ad libitum* access to commercial chow and tap water. Mice received a single intravenous administration of fluorescein-labeled αAC nanofiber (100 μl aliquot of 600 μg/ml) and were then anesthetized with urethane 30 min later. The nucleus of the neurons was stained with DAPI. The fluorescence observations were performed using a laser-scanning confocal microscope (LSM510; Carl Zeiss AG, Oberkochen, Germany). All experimental protocols were performed in accordance with the National Institute of Health Guidelines and animal research of the Neuroscience Society of Japan to minimize the number of animals used and their suffering and the study was approved by the Animal Ethics Committee of Kyoto Institute of Technology. After deep anesthesia with urethane, mice were perfused with PBS (pH 7.4) containing 5 U/ml heparin followed by 4% PFA in PBS (pH 7.4). Brain blocks containing the hippocampus were cryoprotected by 30% sucrose in PBS (pH 7.4) and quickly frozen in Tissue-Tek OCT compound (Sakura Finetechnical, Tokyo, Japan). The sections were obtained by coronal cut on a cryostat (Leica, Wetzlar, Germany) at a thickness of 30 μm. The coverslips were sealed with mounting medium (Vectashield; Vector Laboratories, Burlingame, CA). Confocal images (1,024 x 1,024 pixels) were saved as TIF files by employing Zeiss LSM510 Image-Browser software or Olympus FV10-ASW Ver 1.7 Viewer for Windows and arranged using Photoshop 7.0.

### αAC nanofiber treatment to APPswe/PS1dE9 transgenic mice

APPswe/PS1dE9 Tg mice were obtained from Jackson Laboratory (Bar Harbor, ME) and maintained by crossing Tg mice with B6C3F1 mice. The genotyping for the Tg mice was performed using the PCR method recommended by the Jackson Laboratory. Female Tg mice were selected and used for this study. For intranasal administration test, the solution of αAC nanofiber (6 μl aliquot of 580 μg/ml in PBS (pH 7.4)) was injected twice weekly for 16 weeks (3–7 months of age; *n* = 13). For intravenous administration test, the solution of αAC nanofiber (150 μl aliquot of 820 μg/ml in PBS (pH 7.4)) was injected twice weekly for 8 weeks (5–7 months of age; *n* = 4). At the end of the study, mice were euthanized under anesthesia by the intraperitoneal administration of sodium pentobarbital. The animals were housed four or five per cage in a colony room with a 12 h light (8:00–20:00) /12 h dark (20:00–8:00) cycle and given *ad libitum* access to commercial chow and tap water. All experimental procedures involving mice and their care were conducted in accordance with the ethical guidelines of the Kyoto University Animal Experimentation Committee and the guidelines of the Japanese Pharmacological Society(Approval number: 110025).

### Y-maze test

Spatial memory of the micegiventhe intranasal administration was evaluated according to the Y-maze test. The measurement at the start of the αAC nanofiber treatment, and then 4 and 8 weeks after treatment. Each mouse was placed at one arm of the Y-maze, which has three arms of 30 cm in length with equal angles between all arms (Bio Research Center, Nagoya, Japan). The mice were allowed to move freely within the maze for 8 minutes, and the sequence and the number of arm entries were recorded. A spontaneous alternation behavior, which is used as a measure of spatial memory, was defined as entry into all three arms on consecutive choices. The percentage of spontaneous alternation behavior was calculated as follows; the number of spontaneous alternations/ (the number of total arm entries–2) ×100.

### Morris water maze test

The spatial learning and memory of the mice given the intranasal administration was evaluated at the 16^th^ week of treatment using a Morris water maze according to the traditional method [40]. The Morris water maze, consisting of a circular pool 120 cm in diameter, was filled with water containing 1% skim milk to a depth of 20 cm. A circular platform (12 cm diameter) was placed in the pool. The temperature of the water was kept at 25 ± 2°C. All mice were trained with two trials per day for 5 days consecutively. In each trial, the mice were allowed to swim until they reached the platform or for up to 90 s. If they reached the platform within 90 s, they stayed on it for 10 s. If they could not reach the platform within 90 s, they were moved to the platform and stayed on it for 15 s. On the first day, the platform was placed 2 cm above the surface of the water (visible platform test). From the second to the seventh days, the platform was placed 2 cm below the surface of the water (hidden platform test). The mice swimming in the pool were tracked using a video tracking and analysis system (ACTIMAZE; Actimetrics, Wilmette, IL, USA), and their escape latencies (the time taken to reach the platform) were automatically recorded. The daily latency of each mouse was obtained from the average latencies of the two trials undertaken each day. After each trial, the mice were dried with paper towels before they were returned to the breeding cage.

### Protein extraction from the brain tissue

At the end of treatment, all animals were sacrificed under deep anesthesia with 100-mg/kg sodium pentobarbital (Kyoritsu Seiyaku Corp., Tokyo, Japan) administered intraperitoneally, and the whole brains were removed and used for biochemical or histochemical analysis. These experimental procedures were conducted in accordance with the ethics guidelines of the Kyoto University Animal Experimentation Committee and the guidelines of the Japanese Pharmacological Society. All efforts were made to minimize suffering. For biochemical analysis, the brain tissue was homogenized in five volumes (w/v) of extraction buffer containing 50 mM Tris-HCl (pH 7.5), 5 mM ethylenediaminetetraacetic acid (Nippon Gene, Tokyo, Japan), 1 mM ethylene glycol tetraacetic acid (NacalaiTesque, Inc.), 1% NP-40 (Sigma-Aldrich Corp.), 0.25% deoxycholic acid sodium salt (Sigma-Aldrich Corp.), 0.1 M NaCl, 0.5 mM phenylmethylsulfonyl fluoride (Sigma-Aldrich Corp.), 1 × PhosSTOP (Roche, Basel, Switzerland), and 1% protease inhibitor cocktail (NacalaiTesque, Inc.). The homogenate was centrifuged at 100,000 × g at 4°C for 20 min. The supernatant was collected as a soluble fraction. The pellet was sonicated in 70% formic acid (Wako Pure Chemical Industries, Ltd.), neutralized with 20 volumes of 0.9 M Tris buffer (pH 11.0), and used as an insoluble fraction.

### Enzyme-linked immunosorbent assay for amyloid β

Aβ(1–42) and Aβ (1–40) levels in the brains were measured using an Aβ Enzyme-linked immunosorbent Assay for amyloid-β (ELISA) Kit (Wako Pure Chemical Industries, Inc.) and a microplate reader (Model 680; Bio-Rad Laboratories, Inc.) according to the manufacture’s protocol. The insoluble fractions were diluted 1,000-fold with the dilution buffer provided in the kit. The soluble fractions were diluted 20-fold with the buffer.

### Immunohistochemistry for amyloid β

The brains of mice given the intravenous administration were fixed in 4% paraformaldehyde phosphate buffer solution (Wako Pure Chemical). The fixed tissues were dehydrated with ethanol (Wako Pure Chemical) for 24 h, transferred into xylene (10 min three times), and embedded in paraffin (30 min three times at 60°C). The paraffin-embedded tissue blocks were cut into 4-μm thick sections with a sliding microtome (Nippon Optical Works Co., Ltd., Tokyo, Japan) and extended on Matsunami-Adhesive Silane-coated micro slide glasses (Matsunami Glass Industry, Ltd., Osaka, Japan) at 60°C for 24 h. The sections were deparaffinized with xylene and ethanol (100%, 90%, and 70%). After washing twice in water, 70% formic acid solution was dispensed on to the sections, and they were incubated at room temperature for 5 min. The sections were washed with water (5 min, three times) and 0.2% Tween 20 in PBS (10 min), and then stained with anti-Aβ antibody 6E10 (1:100 dilution; Covance Inc., Princeton, NJ, USA) using a M. O. M.TM Immuno detection Peroxidase Kit (Vector Laboratories Inc., Burlingame, CA, USA), according to the manufacture’s protocol. As a chromogen, 3,3’-diaminobenzidine (Wako Pure Chemical) at a concentration of 0.5 mg/ml in PBS with 0.005% hydrogen peroxide was used. After staining, the sections were washed with water, dehydrated with ethanol (70%, 90%, and 100%) and xylene, and then mounted with mounting medium (Daido Sangyo Co., Ltd., Tokyo, Japan). The sections were observed with a microscope BZ-8100 (Keyence Corp., Osaka, Japan). Aβ plaques in each section were detected using ImageJ software (National Institutes of Health, Bethesda, MD, USA).The number of Aβ plaques in the total section area, the ratio of Aβ plaque area to the whole section area and the average size of Aβ plaque of each section were calculated.

### Data analysis

Data derived from the ELISA was analyzed using Dunnett’s multiple comparison tests. Data derived from the Y-maze and Morris water-maze tests were analyzed using two-way analysis of variance (ANOVA) and the post-hoc Bonferroni test. The software GraphPad Prism (GraphPad Software Inc., San Diego, CA, USA) was used for these analyses, and P < 0.05 was considered statistically significant.

## Supporting information

S1 File (DOCX)

## References

[1] Selkoe DJ. Preventing Alzheimer’s disease. Science.2012; 337: 1488–1492. 10.1126/science.1228541 22997326

[2] Karran E, Mercken M, De Strooper B. The amyloid cascade hypothesis for Alzheimer's disease: an appraisal for the development of therapeutics. Nat. Rev. Drug Discov. 2011; 10: 698–712. 10.1038/nrd3505 21852788

[3] Goedert M, Klug A, Crowther RA. Tau protein, the paired helical filament and Alzheimer's disease. J. Alzheimers. Dis. 2006; 9: 195–207. 10.3233/jad-2006-9s323 16914859

[4] Dobson CM. Protein folding and misfolding. Nature 2003; 426: 884–890. 10.1038/nature02261 14685248

[5] Goto Y, Yagi H, Yamaguchi K, Chatani E, Ban T. Structure, formation and propagation of amyloid fibrils. Curr. Pharm. Des. 2008; 14: 3205–3218. 10.2174/138161208786404146 19075701

[6] Broersen K, Rousseau F, Schymkowitz J. The culprit behind amyloid beta peptide related neurotoxicity in Alzheimer's disease: oligomer size or conformation? Alzheimers Res. Ther. 2010; 2: 12 10.1186/alzrt36 20642866 PMC2949586

[7] Doig AJ. Peptide inhibitors of beta-amyloid aggregation. Curr. Opin. Drug Discov. Devel. 2007; 10: 533–539. 17786851

[8] Parthsarathy V, McClean PL, Hölscher C, Taylor M, Tinker C, Jones G et al A novel retro-inverso peptide inhibitor reduces amyloid deposition, oxidation and inflammation and stimulates neurogenesis in the APPswe/PS1DeltaE9 mouse model of Alzheimer's disease. PLoS ONE 2013; 8: art. no. e54769 10.1371/journal.pone.0054769 23382963 PMC3561363

[9] Kurnellas MP, Brownell SE, Su L, Malkovskiy AV, Rajadas J, Dolganov G et al Chaperone activity of small heat shock proteins underlies therapeutic efficacy in experimental autoimmune encephalomyelitis. J. Biol. Chem. 2012; 287: 36423–36434. 10.1074/jbc.M112.371229 22955287 PMC3476308

[10] Kurnellas MP, Adams CM, Sobel RA, Steinman L, Rothbard JB. Amyloid fibrils composed of hexameric peptides attenuate neuroinflammation. Sci. Transl. Med. 2013; 5: 179ra42 10.1126/scitranslmed.3005681 23552370 PMC3684024

[11] Sreekumar PG, Chothe P, Sharma KK, Baid R, Kompella U, Spee C et al Antiapoptotic properties of alpha-crystallin-derived peptide chaperones and characterization of their uptake transporters in human RPE cells. Inves. Ophthalmol. Vis. Sci. 2013; 54: 2787–2798. 10.1167/iovs.12-11571 PMC3632268 23532520

[12] Clark JI, Muchowski PJ. Small heat-shock proteins and their potential role in human disease. Curr. Opin. Struct. Biol. 2000; 10: 52–59. 10.1016/s0959-440x(99)00048-2 10679464

[13] Horwitz J. Alpha-crystallin. Exp. Eye Res. 2003; 76: 145–153. 10.1016/s0014-4835(02)00278-6 12565801

[14] Fonte V, Kipp DR, Yerg J, Merin D, Forrestal M, Wagner E. et al Suppression of in vivo beta-amyloid peptide toxicity by overexpression of the HSP-16.2 small chaperone protein. J. Biol. Chem. 2008; 283: 784–791. 10.1074/jbc.M703339200 17993648

[15] Dehle FC, Ecroyd H, Musgrave IF, Carver JA. alphaB-Crystallin inhibits the cell toxicity associated with amyloid fibril formation by kappa-casein and the amyloid-beta peptide. Cell Stress Chaperones 2010; 15: 1013–1026. 10.1007/s12192-010-0212-z 20632140 PMC3024074

[16] Tue NT, Shimaji K, Tanaka N, Yamaguchi M. Effect of alphaB-Crystallin on Synthesis and characterization of a peptide identified as a functional element in alphaA-crystallin.Protein Aggregation in Drosophila. J. Biomed. Biotechnol. 2012; 2012: art. no. 252049 10.1155/2012/252049 22505806 PMC3312385

[17] Laganowsky A, Benesch JL, Landau M, Ding L, Sawaya MR, Cascio D et al Crystal structures of truncated alphaA and alphaB crystallins reveal structural mechanisms of polydispersity important for eye lens function. Protein Sci. 2010; 19: 1031–1043. 10.1002/pro.380 20440841 PMC2868245

[18] Sharma KK, Kumar RS, Kumar GS, Quinn PT. J. Biol. Chem. 2000; 275: 3767–3771. 10.1074/jbc.275.6.3767 10660525

[19] Santhoshkumar P, Sharma KK. Inhibition of amyloid fibrillogenesis and toxicity by a peptide chaperon. Mol. Cell Biochem. 2004; 267: 147–155. 10.1023/b:mcbi.0000049373.15558.b8 15663196

[20] Bhattacharyya J, Padmanabha Udupa EG, Wang J, Sharma KK. Mini-alphaB-crystallin: a functional element of alphaB-crystallin with chaperone-like activity. Biochemistry 2006; 45: 3069–3076. 10.1021/bi0518141 16503662 PMC2615690

[21] Santhoshkumar P, Raju M, Sharma KK. αA-crystallin peptide 66SDRDKFVIFLDVKHF80 accumulating in aging lens impairs the function of α-crystallin and induces lens protein aggregation. PLoS ONE 2011; 6:art. no. e19291 10.1371/journal.pone.0019291 21552534 PMC3084282

[22] Raju M, Santhoshkumar P, Xie L, Sharma KK. Addition of αA-crystallin sequence 164–173 to a mini-chaperone DFVIFLDVKHFSPEDLT alters the conformation but not the chaperone-like activity. Biochemistry 2014; 53: 2615–2623. 10.1021/bi4017268 24697516 PMC4007981

[23] Santhoshkumar P, Karmakar S, Sharma KK. Alpha-crystallin-derived peptides as therapeutic chaperones. Biochim. Biophys. Acta 2016; 1860: 246–251. 10.1016/j.bbagen.2015.06.010 26141743 PMC4673008

[24] Raju M, Santhoshkumar P, Sharma KK. Cell-penetrating chaperone peptide prevents protein aggregation and protects against cell apoptosis. Adv. Biosyst. 2018; 2:art. no.1700095 10.1002/adbi.201700095 30271873 PMC6157914

[25] Ghosh JG, Estrada MR, Clark JI. Interactive domains for chaperone activity in the small heat shock protein, human alphaB crystallin. Biochemistry 2005; 44: 14854–14869. 10.1021/bi0503910 16274233

[26] Nahomi RB, Wang B, Raghavan CT, Voss O, Doseff AI, Santhoshkumar P et al Chaperone peptides of α-crystallin inhibit epithelial cell apoptosis, protein insolubilization, and opacification in experimental cataracts. J. Biol. Chem. 2013; 288: 13022–13035. 10.1074/jbc.M112.440214 23508955 PMC3642345

[27] Banerjee PR, Pande A, Shekhtman A, Pande J. Molecular mechanism of the chaperone function of mini-α-crystallin, a 19-residue peptide of human α-crystallin. Biochemistry 2015; 54:505–515. 10.1021/bi5014479 25478825 PMC4303307

[28] Tanaka N, Tanaka R, Tokuhara M, Kunugi S, Lee YF, Hamada D. Amyloid fibril formation and chaperone-like activity of peptides from alphaA-crystallin. Biochemistry 2008; 47: 2961–2967. 10.1021/bi701823g 18232642

[29] Fukuhara S, Nishigaki T, Miyata K, Tsuchiya N, Waku T, Tanaka N. Mechanism of the chaperone-like and antichaperone activities of amyloid fibrils of peptides from alphaA-crystallin. Biochemistry 2012; 51: 5394–5401. 10.1021/bi3004236 22694216

[30] Okuyama K, Nishiura C, Mizushima F, Minoura K, Sumida M, Taniguchi T, et al Linkage-dependent contribution of repeat peptides to self-aggregation of three- or four-repeat microtubule-binding domains in tau protein. FEBS J. 2008; 275: 1529–1539. 10.1111/j.1742-4658.2008.06312.x 18312411

[31] Friedhoff P, von Bergen M, Mandelkow EM, Davies P, Mandelkow E. A nucleated assembly mechanism of Alzheimer paired helical filaments. Proc. Natl. Acad. Sci. U S A 1998; 95: 15712–15717. 10.1073/pnas.95.26.15712 9861035 PMC28109

[32] Joliot A, Prochiantz A. Transduction peptides: from technology to physiology. Nat. Cell Biol. 2004; 6: 189–196. 10.1038/ncb0304-189 15039791

[33] Popiel HA, Nagai Y, Fujikake N, Toda T. Delivery of the aggregate inhibitor peptide QBP1 into the mouse brain using PTDs and its therapeutic effect on polyglutamine disease mice. Neurosci. Lett. 2009; 449: 87–92. 10.1016/j.neulet.2008.06.015 18603372

[34] Kamei N., Takeda-Morishita M. Brain delivery of insulin boosted by intranasal coadministration with cell-penetrating peptides.J. Control. Release 2015; 197: 105–110. 10.1016/j.jconrel.2014.11.004 25445695

[35] Ban T, Hamada D, Hasegawa K, Naiki H, Goto Y. Direct observation of amyloid fibril growth monitored by thioflavin T fluorescence. J. Biol. Chem. 2003; 278: 16462–16465. 10.1074/jbc.C300049200 12646572

[36] Morita S, Miyata S. Different vascular permeability between the sensory and secretory circumventricular organs of adult mouse brain. Cell. Tissue Res. 2012; 349: 589–603. 10.1007/s00441-012-1421-9 22584508

[37] Khan AA, Mao XO, Banwait S, Jin K, Greenberg DA. Neuroglobin attenuates beta-amyloid neurotoxicity in vitro and transgenic Alzheimer phenotype in vivo. Proc. Natl. Acad. Sci. U S A 2007; 104: 19114–19119. 10.1073/pnas.0706167104 18025470 PMC2141917

[38] Dhuria SV, Hanson LR, Frey II WH. Intranasal delivery to the central nervous system: mechanisms and experimental considerations. J. Pharma. Sci. 2010; 99: 1654–1673. 10.1002/jps.21924 19877171

[39] Kanazawa T, Akiyama F, Kakizaki S, Takashima Y, Seta Y. Delivery of siRNA to the brain using a combination of nose-to-brain delivery and cell-penetrating peptide-modified nano-micelles. Biomaterials 2013; 34: 9220–9226. 10.1016/j.biomaterials.2013.08.036 23992922

[40] Vorhees CV, Williams MT. Morris water maze: procedures for assessing special and related forms of learning and memory. Nat. Protoc. 2006; 1:848–858. 10.1038/nprot.2006.116 17406317 PMC2895266

[41] Konno T. Amyloid-induced aggregation and precipitation of soluble proteins: an electrostatic contribution of the Alzheimer's beta(25–35) amyloid fibril. Biochemistry 2001; 40: 2148–2154. 10.1021/bi002156h 11329283

[42] Olzscha H, Schermann SM, Woerner AC, Pinkert S, Hecht MH, Tartaglia GG et al Amyloid-like aggregates sequester numerous metastable proteins with essential cellular functions. Cell 2011; 144: 67–78. 10.1016/j.cell.2010.11.050 21215370

[43] Waku T, Kitagawa Y, Kawabata K, Nishigaki S, Kunugi S, & Tanaka N. Self-assembled β–σηεετ πεπτιδε νανοϕιβερσ ϕορ εϕϕιχιεντ αντιγεν δελιϖερψ. Chem. Lett. 2013; 42: 1441–1443.

[44] Minami T, Matsumoto S, Sanada Y, Waku T, Tanaka N, Sakurai K. Rod-like architecture and cross-sectional structure of an amyloid protofilament-like peptide supermolecule in aqueous solution.Polym. J. 2016; 48: 197–202.

[45] Dietz GP, Bahr M. Peptide-enhanced cellular internalization of proteins in neuroscience. Brain Res. Bull. 2005; 68: 103–114. 10.1016/j.brainresbull.2005.08.015 16325010

[46] Herve F, Ghinea N, Scherrmann JM. CNS delivery via adsorptive transcytosis. AAPS J. 2008; 10: 455–472. 10.1208/s12248-008-9055-2 18726697 PMC2761699

[47] Mazza M, Notman R, Anwar J, Rodger A, Hicks M, Parkinson G et al Nanofiber-based delivery of therapeutic peptides to the brain. ACS nano 2013; 7: 1016–1026. 10.1021/nn305193d 23289352

[48] Ono K, Yoshiike Y, Takashima A, Hasegawa K, Naiki H, Yamada M. Potent anti-amyloidogenic and fibril-destabilizing effects of polyphenols in vitro: implications for the prevention and therapeutics of Alzheimer's disease. J. Neurochem. 2003; 87: 172–181. 10.1046/j.1471-4159.2003.01976.x 12969264

[49] Yang F, Lim GP, Begum AN, Ubeda OJ, Simmons MR, Ambegaokar SS et al Curcumin inhibits formation of amyloid beta oligomers and fibrils, binds plaques, and reduces amyloid in vivo. J. Biol. Chem. 2005; 280: 5892–5901. 10.1074/jbc.M404751200 15590663

[50] Hattori M, Sugino E, Minoura K, In Y, Sumida M, Taniguchi T et al Different inhibitory response of cyanidin and methylene blue for filament formation of tau microtubule-binding domain. Biochem. Biophys. Res. Commun. 2008; 374: 158–163. 10.1016/j.bbrc.2008.07.001 18619417

[51] Aisen PS, Gauthier S, Ferris SH, Saumier D, Haine D, Garceau D et al Tramiprosate in mild-to-moderate Alzheimer’s disease–a randomized, double-blind, placebo-controlled, multi-centre study (the Alphase Study). Arch. Med. Sci. 2011; 7:102–111. 10.5114/aoms.2011.20612 22291741 PMC3258678

[52] Salloway S, Sperling R, Keren R, Porsteinsson AP, Van Dyck CH, Tariot PN et al A phase 2 randomized trial of ELND005, scyllo-inositol, in mild to moderate Alzheimer disease. Neurology 2011; 77: 1253–1262. 10.1212/WNL.0b013e3182309fa5 21917766 PMC3179648

[53] Matlack KE, Tardiff DF, Narayan P, Hamamichi S, Caldwell KA, Caldwell GA et al Clioquinol promotes the degradation of metal-dependent amyloid-β (Aβ) oligomers to restore endocytosis and ameliorate Aβ toxicity. Proc. Natl. Acad. Sci. U S A 2014; 111:4013–4018. 10.1073/pnas.1402228111 24591589 PMC3964050

[54] Ryan TM, Roberts BR, McColl G, Hare DJ, Doble PA, Li QX et al Stabilization of nontoxic Aβ-oligomers: insights into the mechanism of action of hydroxyquinolines in Alzheimer's disease. J. Neurosci. 2015; 35: 2871–2884. 10.1523/JNEUROSCI.2912-14.2015 25698727 PMC6605585

[55] Wang Z, Wang Y, Li W, Mao F, Sun Y, Huang L et al Design, synthesis, and evaluation of multitarget-directed selenium-containing clioquinol derivatives for the treatment of Alzheimer’s disease. ACS Chem. Neurosci. 2014; 5: 952–962. 10.1021/cn500119g 25121395

[56] Yang H, Qu T, Yang H, Wei L, Xie Z, Wang P et al Self-assembling nanofibers improve cognitive impairment in a transgenic mice model of Alzheimer's disease. Neurosci. Lett. 2013; 556: 63–68. 10.1016/j.neulet.2013.09.063 24103374

[57] Kurnellas MP, Ghosn EEB, Schartner JM, Baker J, Rothbard JJ, Negrin RS et al Amyloid fibrils activate B-1a lymphocytes to ameliorate inflammatory brain disease Proc. Natl. Acad. Sci. U S A 2015; 112:15016–15023. 10.1073/pnas.1521206112 26621719 PMC4679000

[58] Kurnellas MP, Schartner JM, Fathman CG, Jagger A, Steinman L, Rothbard JB. Mechanisms of action of therapeutic amyloidogenic hexapeptides in amelioration of inflammatory brain disease. J. Exp. Med. 2014; 211:1847–1856. 10.1084/jem.20140107 25073790 PMC4144739

[59] Sharoar MG, Thapa A, Shahnawaz M, Ramasamy VS, Woo ER, Shin SY, Park IS. Keampferol-3-O-rhamnoside abrogates amyloid beta toxicity by modulating monomers and remodeling oligomers and fibrils to non-toxic aggregates. J. Biomedi. Sci. 2012;19, 104. 10.1186/1423-0127-19-104 PMC3541263 23259691

[60] Thapa A, Woo ER, Chi EY, Sharoar MG, Jin HG, Shin SY, Park IS. Biflavonoids are superior to monoflavonoids in inhibiting amyloid-β toxicity and fibrillogenesis via accumulation of nontoxic oligomer-like structures. Biochemistry 2011; 50, 2445–2455. 10.1021/bi101731d 21322641

[61] Yao TM, Tomoo K, Ishida T, Hasegawa H, Sasaki M, Taniguchi T. Aggregation analysis of the microtubule binding domain in tau protein by spectroscopic methods. J. Biochem. 2003; 134: 91–99. 10.1093/jb/mvg116 12944375

[62] Sakono M, Zako T, Ueda H, Yohda M, Maeda M. Formation of highly toxic soluble amyloid beta oligomers by the molecular chaperone prefoldin. FEBS J. 2008; 275: 5982–5983. 10.1111/j.1742-4658.2008.06727.x 19021772

