# Supplementary material for: Development of a brain-permeable peptide nanofiber that prevents aggregation of Alzheimer pathogenic proteins
Source: PLoS One. 2020 Jul 24;15(7):e0235979. doi: 10.1371/journal.pone.0235979 (PMC7380640; doi:10.1371/journal.pone.0235979)
Supplement: S1 File — (DOCX) [file pone.0235979.s001.docx]

**Supporting information**

**Development of a brain-permeable peptide nanofiber that prevents aggregation of Alzheimer pathogenic proteins**

Naoki Tanaka^1$^, Michiaki Okuda^3^, Tatsutoshi Nishigaki^1^, Nobuhiko Tsuchiya^1^, Yukako Kobayashi^1^, Takuya Uemura^1^, Sayaka Kumo^1^, Hachiro Sugimoto^4^, Seiji Miyata^2^ and Tomonori Waku^1^*

^1^Faculty of Molecular Chemistry and Engineering, ^2^Faculty of Applied Biology,

Kyoto Institute of Technology, Matsugasaki, Sakyo, Kyoto 606-8585, Japan

^3^Department of Pharmacology, Graduate School of Pharmaceutical Sciences, Kyoto University

46-29 Yoshida-Shimo-Adachi-cho, Sakyo, Kyoto 606-8501, Japan

^4^Faculty of Life and Medical Sciences, Doshisha University

1-3 Tatara Miyakodani, Kyotanabe, Kyoto 610-0394, Japan

^$^Deceased 13th November 2018.

*Corresponding author: Tomonori Waku

TEL: +81-75-724-7811 FAX: +81-75-724-7800 E-mail: [waku1214@kit.ac.jp](mailto:waku1214@kit.ac.jp)

**Key words:** Alzheimer’s disease, amyloid, peptide nanofiber, neurotoxicity


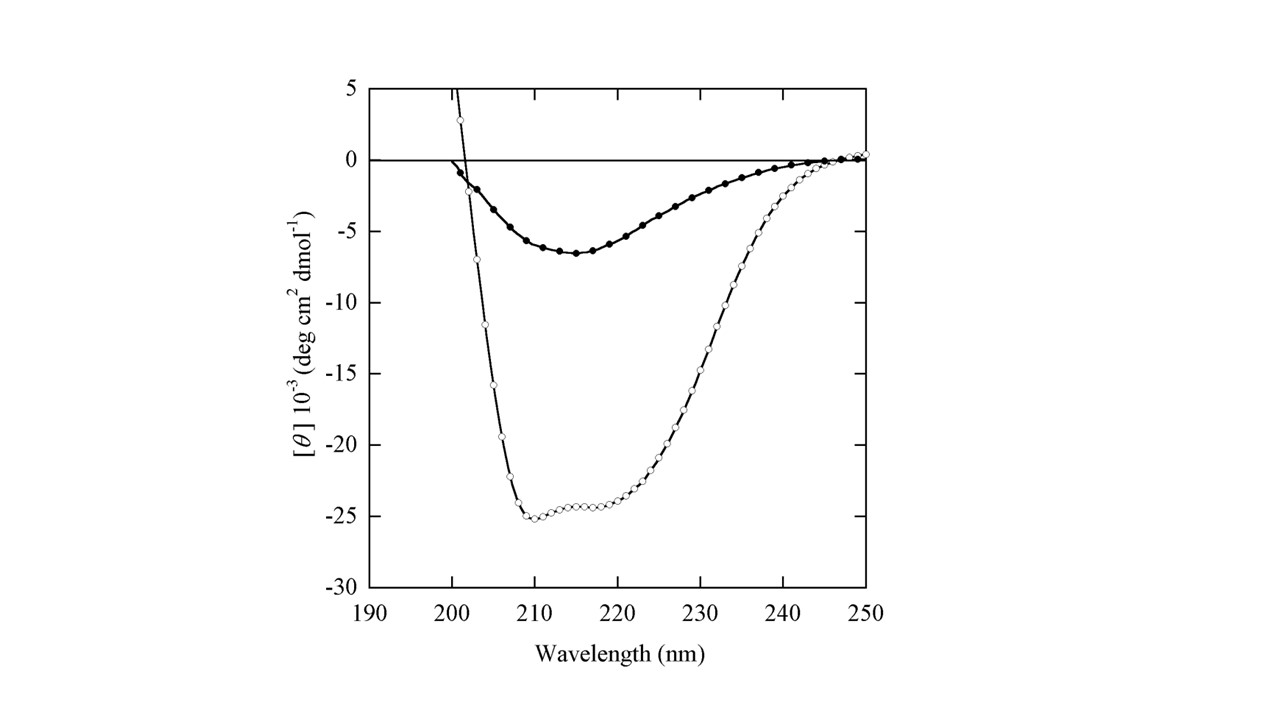


**Figure S1.** Spectroscopic characterization of the αAC(71-88)Antp nanofiber. The secondary structure of αAC(71-88)Antp and its fibril form were monitored by far-UV CD spectroscopy at 25^o^C. Open circle, CD spectrum of αAC(71-88)Antp; closed circle, CD spectrum of the αAC(71-88)Antp nanofiber prepared after 24 h incubation at 60^o^C. The peptide concentration was 0.2 mg/ml (46 μM). The buffer solution used for these experiments was 5 mM sodium phosphate (pH 7.5), 100 mM NaCl, 10% HFIP.


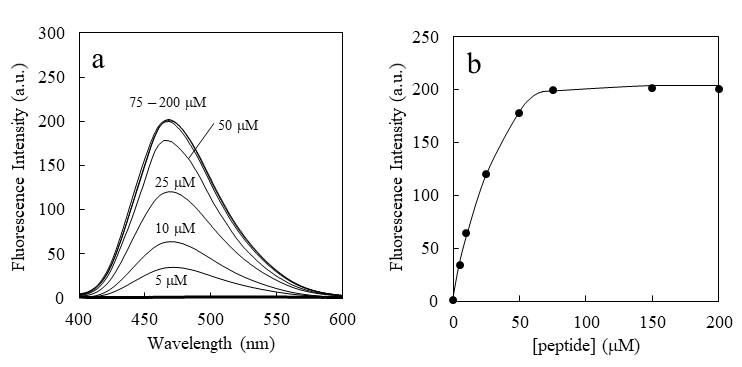


**Figure S2.**  (a) The fluorescence spectra of ANS in the presence of various concentrations of αAC(71-88)Antp nanofiber. The concentration shown in the figure is the molar concentration of αAC(71-88)Antp peptide. Fluorescence spectra of 20 μM ANS were obtained with excitation at 350 nm. (b) Change in the fluorescence intensity of 20 μM ANS at 465 nm as the concentration of αAC(71-88)Antp nanofiber increase. The concentration of the nanofibers were shown as the peptide concentrations.

**Figure S3.** Time course of ThT fluorescence intensity of nanofibers dispersions incubated at 37^o^C. Blue triangle plot, 100 μg/mL of the αAC(71-88)Antp nanofiber; blue circle plot, 44 μg/mL of the αAC(71-88)Antp nanofiber; black circle plot, 11 μg/mL of αAC nanofiber; red triangle plot, 30 μg/mL of the neutral αAC nanofiber; red circle plot, 13 μg/mL of the neutral αAC nanofiber. Each measurement was repeated three times. The plots are the averaged value (mean ± SD). The concentration of ThT was 20 μM.


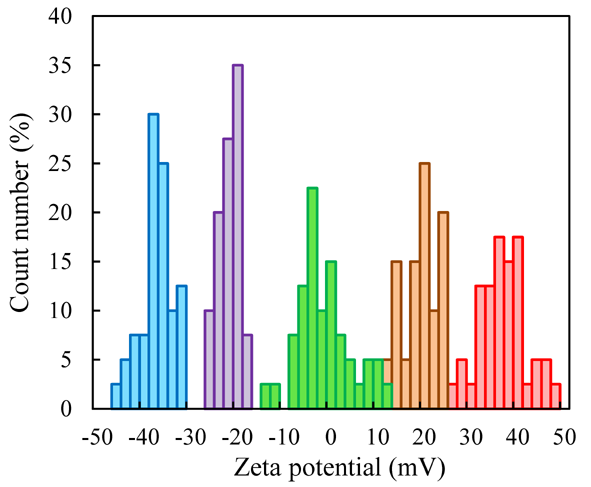


**Figure S4.** Distribution of zeta potential values of the αAC nanofibers calculated from their electrophoretic mobility. The αAC nanofibers were prepared from a solution mixture of various molar ratios of peptides. αAC(71-88):αAC(71-88)Antp ratios were 1:0 (blue), 10:1 (purple), 4:1 (green), 2:1 (orange) and 0:1 (red).

**Figure S5.** MALDI TOF mass spectra of the peptide fragments of αAC peptides and the neutral αAC nanofiber after incubation with trypsin. The molecular weights assigned to the peptide fragments of αAC peptide are shown in the red boxes. The molecular weight of intact αAC(71-88) is shown in the blue box.


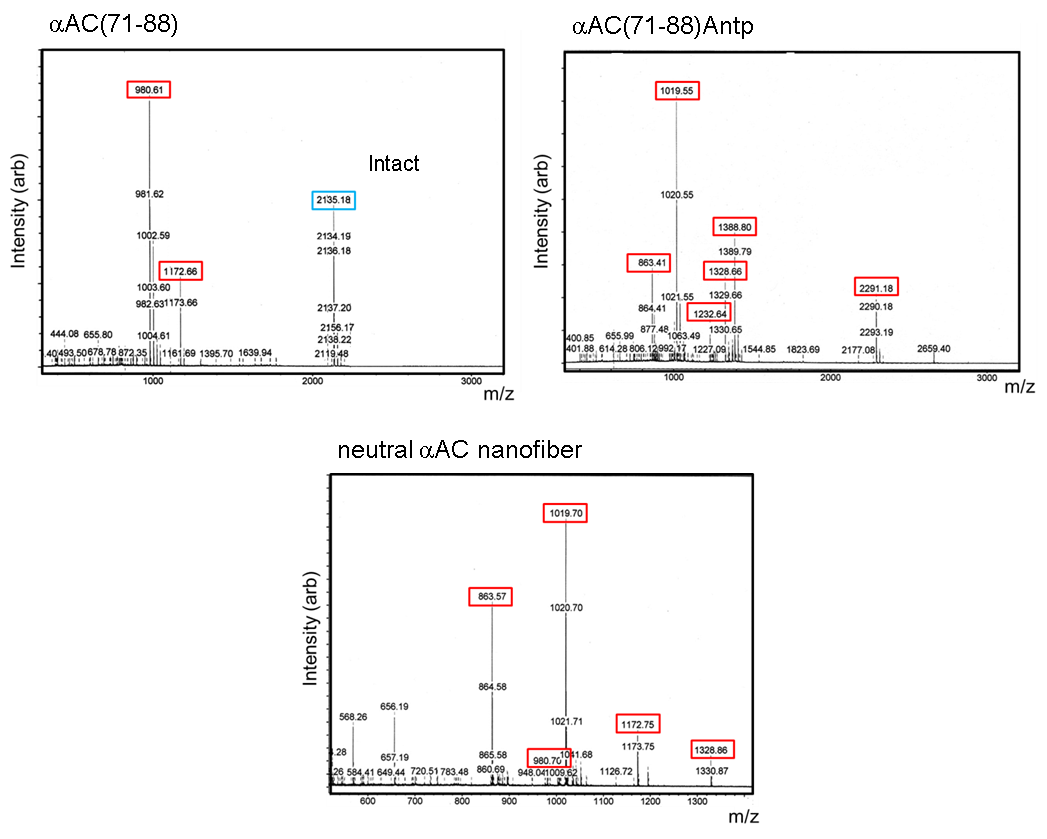


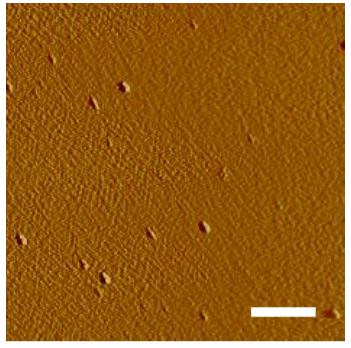


**Figure S6.** AFM image of nanofibers obtained using the freeze-thaw procedure from a solution mixture of αAC(71-88) and αAC(71-88)Antp (molar ratio = 2:1). The arrows represent nanofibers. The scale bar represents 200 nm.


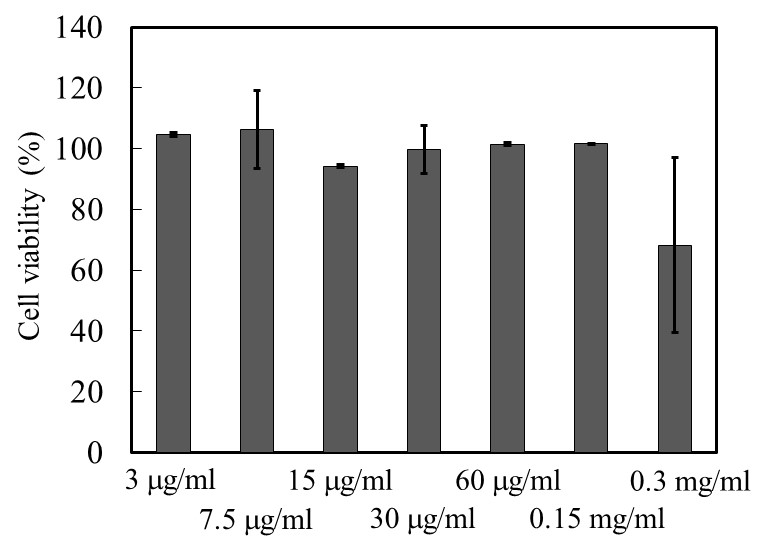


**Figure S7.** Cytotoxicity assay of the neutral αAC nanofibers f obtained using the freeze-thaw procedure from a solution mixture of αAC(71-88) and αAC(71-88)Antp (molar ratio = 2:1)　against PC12 cells using the LDH assay.


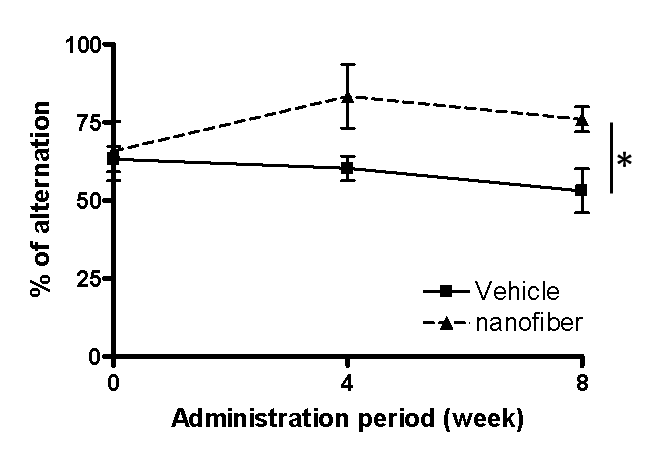


**Figure S8.** Effect of treatment with neutral αAC nanofibers on APPswe/PS1dE9 transgenic AD model mice. Percentage of spontaneous alternation behavior in the Y-maze test of mice administered αAC nanofibers intravenously. The neutral αAC nanofibers were intravenously administered to female, 5-month old mice at ca. 3 mg/kg twice weekly for 8 weeks. The open and closed circle plots indicate the vehicle (n = 4) and neutral αAC nanofiber group (n = 4), respectively. The percentages of spontaneous alternation behavior are indicated by mean ± SEM. *, p < 0.05 by two-way RM ANOVA.

**
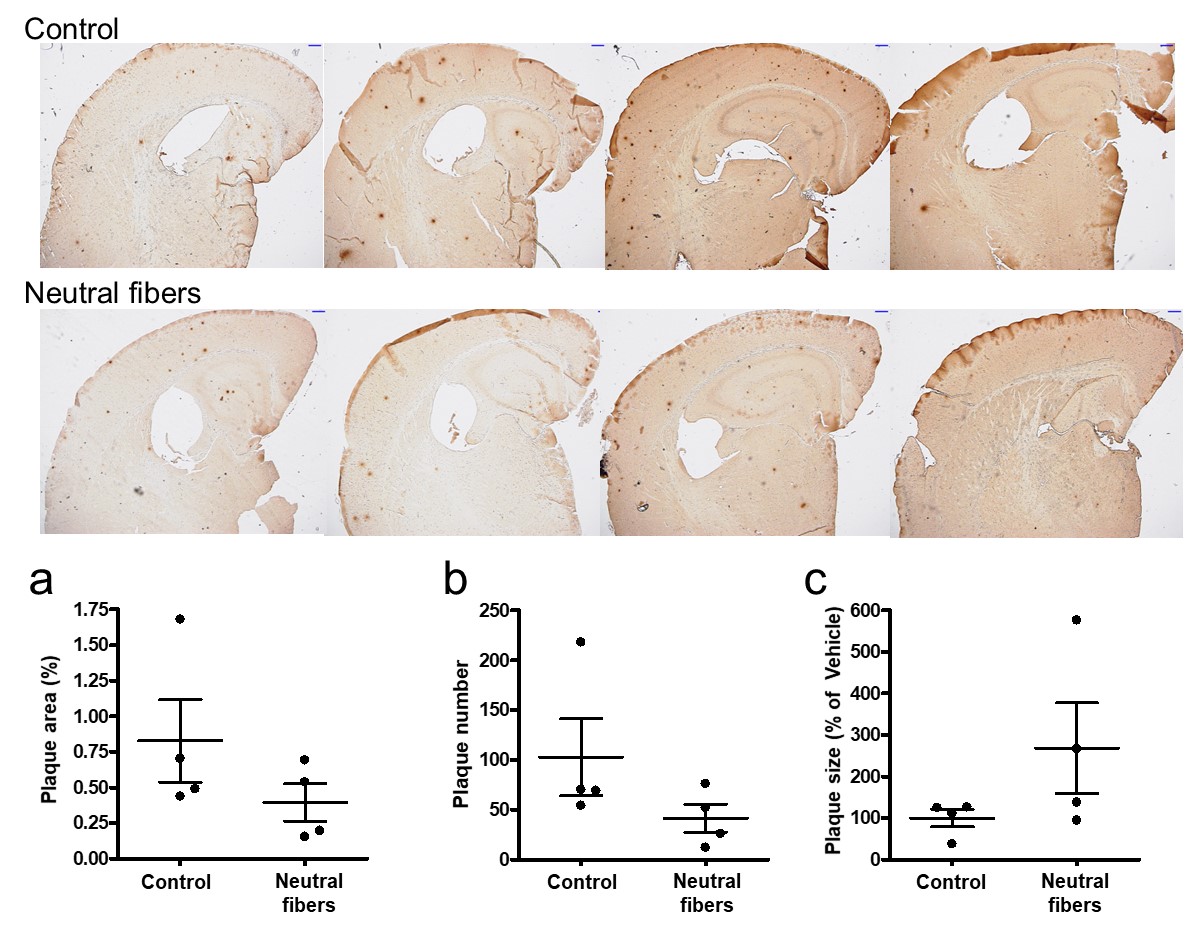
**

**Figure S9.** Immunohistochemical analysis of the brain Aβ. Brain sections were immunostained with anti-Aβ antibody 6E10. (a) Percentage of the area of Aβ plaques to the total area of brain sections. (b) the number of Aβ plaques in the brain sections, (c) the average size of Aβ plaques in the brain sections. n = 4. Data are shown as mean ± SEM.
